# Molecular analysis of long COVID and new-onset diabetes mellitus: pathobiological relationships and current mechanistic views

**DOI:** 10.3389/fendo.2025.1737894

**Published:** 2025-12-18

**Authors:** Getasew Shitaye, Muluabay Getie, Zewdie Mekonnen, Gianluca D’Abrosca, Roberto Fattorusso, Carla Isernia, Asmare Amuamuta, Gaetano Malgieri

**Affiliations:** 1 Department of Environmental, Biological and Pharmaceutical Science and Technology, University of Campania “Luigi Vanvitelli”, Caserta, Italy; 2 Department of Biomedical sciences, College of Medicine and Health Sciences, Bahir Dar University, Bahir Dar, Ethiopia; 3 Department of Biochemistry, School of Medicine, College of Health Sciences, Addis Ababa University, Addis Ababa, Ethiopia; 4 Department of Human Science, Link Campus University, Roma, Italy

**Keywords:** SARS-CoV-2, viral RNA persistence, ACE2/RAS pathway, metabolic disturbance, new-onset diabetes, insulin resistance, pancreatic β-cell, long COVID

## Abstract

Long COVID, or post-acute sequelae of severe acute respiratory syndrome coronavirus 2 (SARS-CoV-2) infection (PASC), refers to a range of persistent health effects associated with SARS-CoV-2 infection. Long COVID is a complex, multisystem disorder that can affect nearly every organ system and is strongly linked with the incidence of diabetes and other chronic conditions. Increasing evidence also connects persistent SARS-CoV-2 infection with the development of new-onset diabetes and other metabolic disorders. In this review, we assess the current evidence and discuss the incidence of new-onset diabetes, along with the pathobiological mechanisms by which SARS-CoV-2 may contribute to the progression of both new-onset type 1 and type 2 diabetes mellitus (T1DM and T2DM). We summarize the latest understanding of the molecular and cellular mechanisms underlying SARS-CoV-2–associated new-onset diabetes. Potential mechanisms include direct damage to pancreatic β-cells, inflammation, insulin resistance, and autoimmune responses. Dysregulation of the ACE2/renin–angiotensin system (RAS) pathway has been linked to multiple inter-organ pathologies, and increased inflammatory cytokines together with dysregulation of interferon regulatory factors (IRFs)—such as overexpression of IRF1—appear to represent key mechanistic links to widespread tissue damage and metabolic alterations. Moreover, the presence of viral RNA or viral RNA fragments may directly damage pancreatic islets, contributing to insulin resistance and β-cell dysfunction that, in turn, may promote the development of new-onset diabetes. In light of these findings, this review further examines evidence supporting the persistence of SARS-CoV-2 RNA in PASC reservoir tissues, including the pancreas, and its potential association with the development of new-onset diabetes mellitus.

## 1 Introduction

SARS-CoV-2, which emerged as a novel coronavirus in early December 2019, caused the mortal global pandemic COVID-19 (1). It is a highly contagious virus that causes acute infections with various clinical phenotypes, ranging from asymptomatic infection to life-threatening COVID-19. It can lead to multiple organ failure and severe pulmonary and extrapulmonary manifestations characterized by extreme inflammation and cytokine storm (2–4). Beyond the health impacts, COVID-19 has devastated individual lives, health systems, and global economies (5).

SARS-CoV-2 exhibits highly efficient human-to-human transmission, strong signs of presymptomatic and asymptomatic spread, and incubation periods that differ from those of previous coronavirus outbreaks such as SARS-CoV and MERS. More importantly, SARS-CoV-2 shows dynamic antigenic landscapes, continuously evolving to generate new variants with increased transmissibility and host immune evasion (6–8).

Indeed, SARS-CoV-2 is continuously evolving (9), and even common subvariants adapt to human cells and modify their mechanisms of entry (10). Evidence suggests that humans will coexist with this virus for a long time. For instance, a recent study reported that high-risk viruses, including coronaviruses, are already circulating in wild and farm animals (11). Another study analyzing human and non-human genomic data revealed that domestic animals such as dogs, cats, mink, and white-tailed deer carried SARS-CoV-2 strains clustering closely with human-origin viruses (12), implicating the possibility of emergent novel variants that could trigger COVID-19 re-emergence. Thus, the possibility of future spillovers involving unknown coronaviruses and the emergence of variants of concern requires advancing our knowledge of molecular mechanisms of virus replication and virus–host interactions. In addition to metabolic abnormalities such as hyperlipidemia and cardiovascular, gastrointestinal, and neurologic conditions, growing evidence indicates the incidence of diabetes as a post-acute sequela following COVID-19 pandemic (5, 13–16). Recent studies underscore an increased risk of both, type 1 and 2, DM after SARS-CoV-2 infection also in vaccinated individuals (17, 18). However, consistent data on the causal association and mechanistic links of new-onset diabetes mellitus (NODM) in long COVID remain scarce. The detailed molecular basis of NODM progression following SARS-CoV-2 infection also remains poorly understood.

Therefore, beyond reviewing the current knowledge on new-onset diabetes, the principal aim of this review is to assess current evidence and discuss the incidence of new-onset diabetes mellitus and the pathobiological mechanistic links by which SARS-CoV-2 may contribute to the progression of new-onset type 1 and type 2 diabetes mellitus (T1DM and T2DM). First, we summarize information regarding long COVID and SARS-CoV-2 genome organization and replication mechanisms. Then, focusing on pancreatic tissue, we summarize the gene expression profiles related to SARS-CoV-2 entry along with host cell receptors that may provide mechanistic links between SARS-CoV-2 infection, pancreatic islets, and inter-organ pathobiological pathways in the progression of new-onset DM. We also highlight the incidence of diabetes mellitus in the post-acute sequelae of SARS-CoV-2 infection. Finally, we review evidence supporting the persistence of SARS-CoV-2 RNA in post-acute sequelae of SARS-CoV-2 infection (PASC) reservoir tissues and its potential association with the development of new-onset DM.

### 1.1 Overview of mechanisms of long COVID

The term long COVID, or postacute sequelae of SARS-CoV-2 infection (PASC), refers to as the association of SARS-CoV-2 infection with persistent health effects, with new symptoms occurring or relapsing after four weeks of acute infection (19). Although definitions of long COVID vary and many individuals report unexplained symptoms, research shows that 0–35% of COVID-19 survivors experience ongoing symptoms such as fatigue, cognitive impairment, or mental health issues including posttraumatic stress disorder (PTSD) and anxiety (20–22). Despite millions continuing to suffer from long COVID (5, 23), no treatments with proven efficacy and no diagnostic tests or therapeutic solutions are currently available.

Long COVID is a complex, multisystem disorder affecting nearly every organ in the body, including the endocrine system (24, 25), cardiovascular system (15, 24), nervous system (25, 26), gastrointestinal system (27), musculoskeletal system (28), immune system (5).

Although there are potentially conflicting data, research has indicated sex differences in acute COVID-19 and long COVID outcomes. For instance, some authors reported that, during acute infection, males experience greater disease severity and mortality, whereas a larger percentage of females develop long COVID (21). Furthermore, epidemiological evidence and retrospective studies documented that COVID-19 was more likely to affect older males with comorbidities such as malignant tumors and diseases of the endocrine, digestive, respiratory, cardiovascular, cerebrovascular, and nervous systems (29, 30).

Such differences can be explained in terms of innate and adaptive immune responses that vary by sex. For example, Hamlin et al. uncovered multiple sex-specific immune pathways associated with long COVID (31). In pediatric patients, long COVID can cause unexpected health consequences such as multisystem inflammatory syndrome in children (MIS-C) (32, 33).

The host immune response at the time of acute illness plays a significant role in the pathogenesis of COVID-19. Marked immune dysregulation, including elevated expression of inflammatory mediators, is identified in acute-phase infections (34, 35). Similarly, researchers have discovered genes associated with coagulopathy, lung fibrosis, multi-organ damage, and long COVID-19 (36, 37).

**Figure 1:**
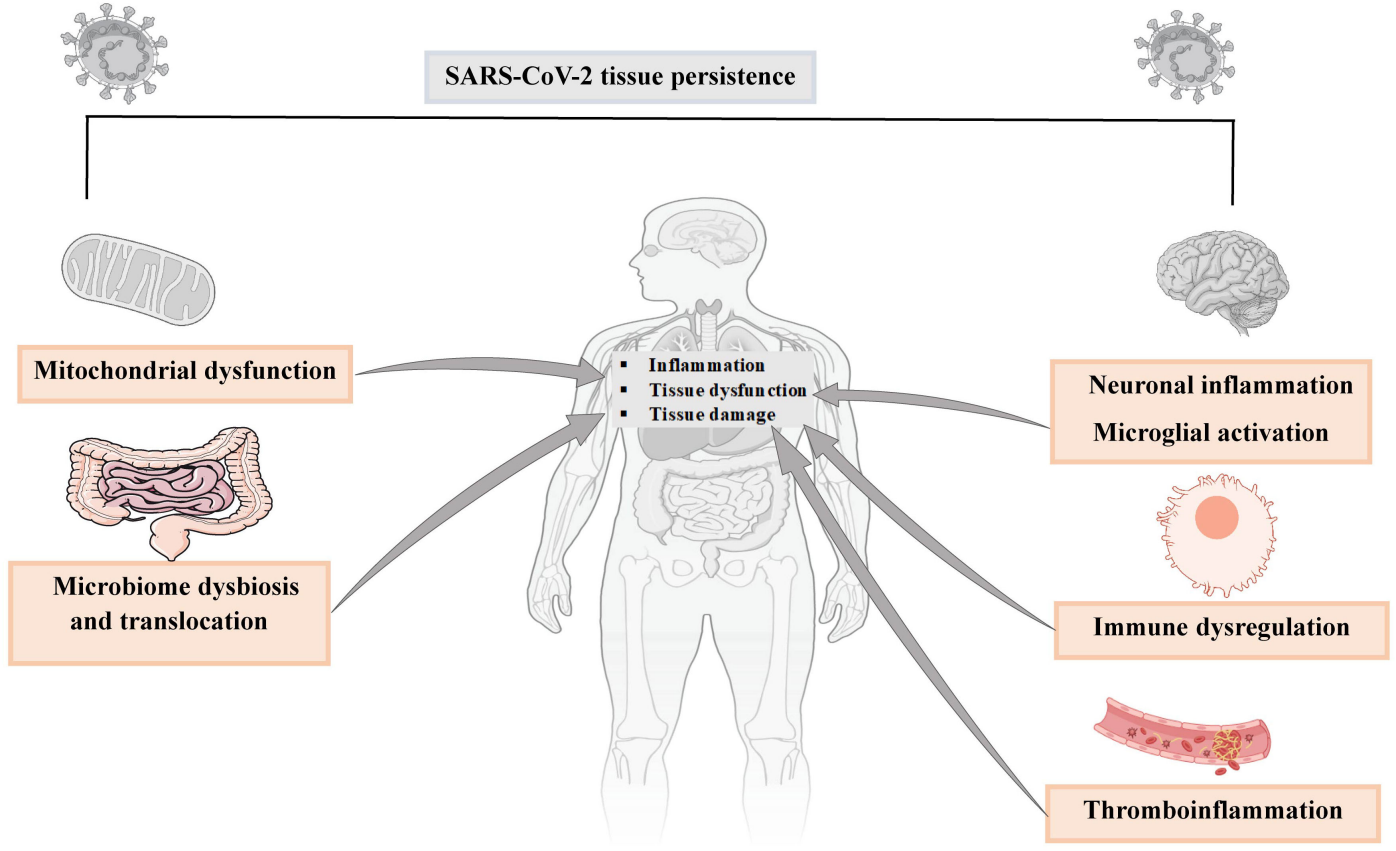
Mechanisms of long COVID. The figure partially explains viral persistence in tissues and related clinical manifestations of long COVID (5). Parts of the figure were drawn and adapted from Servier Medical Art (http://smart.servier.com), licensed under CC BY 4.0 (https://creativecommons.org/licenses/by/4.0/).

It is now established that long COVID is not confined to respiratory tissues. Over 200 symptoms have been observed across nearly all organ systems in the human body (38), evidencing the complexity of this multisystem disease. Extensive research using immunological, transcriptomic, clinical, and virological data shows that the mechanisms of long COVID are largely non-exclusive. They may involve host cell dysfunction, sex-specific immune dysregulation, and persistent inflammation. For example, excessive immune activation may lead to several downstream pathologies, such as mitochondrial dysfunction, metabolic dysfunction, excessive inflammatory responses, neuronal inflammation, microglial activation, and other abnormalities (**Figure 1**). As a consequence, multiorgan damage may occur with diverse clinical manifestations. Collectively, viral antigen persistence and immune dysregulation are two major mechanisms that might drive long COVID (38–43).

Indeed, the persistence of SARS-CoV-2 RNA in different tissues is strongly associated with the development of long COVID (44). For these reasons, tissue viral persistence could lead to long-term immunological perturbations, neurodegenerative diseases, and other chronic health complications (45).

### 1.2 SARS-CoV-2 genome organization and replication

**Figure 2:**
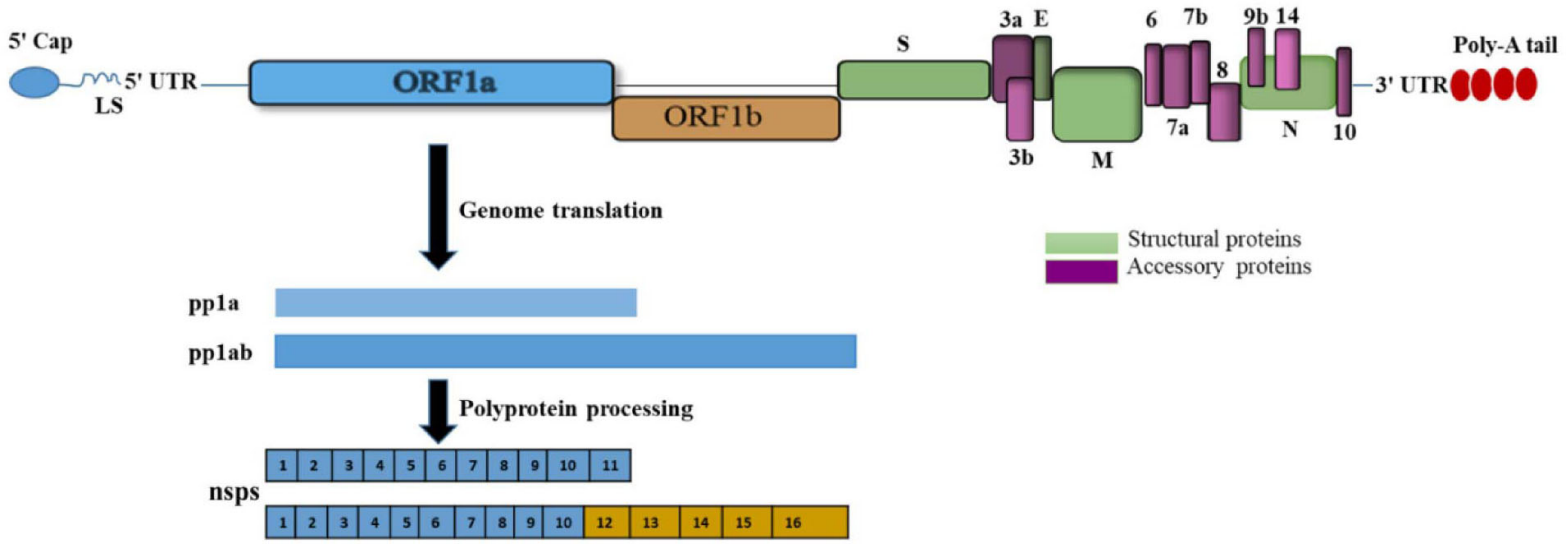
Scheme illustrating SARS-CoV-2 genome organization: SARS-CoV-2 encompasses mRNA capped at the 5′ end, a leader sequence (LS), poly-A tail at 3′ end, and 5′ and 3′ UTR. Its genes ORF1a, ORF1b, are translated into polyproteins (pp1a and pp1ab), respectively. It also comprises structural proteins (i.e. S: spike, E: envelope, M: membrane, and N: nucleocapsid), and several accessory proteins: ORF3a, ORF3b, ORF6, ORF7a, ORF7b, ORF8, ORF9b, ORF14, and ORF10.

Viruses of the family Coronaviridae share the same general genome organization of a single-stranded, positive-sense RNA genome with length ranges from 26 to 32 kilobases (46, 47). Thus, SARS-CoV-2 is an enveloped, positive sense, single-stranded RNA virus, that shares (about 79%) sequence identity with SARS-CoV-1 in their full-length genome sequences (48, 49). SARS-CoV-2 RNA (+) genome encodes at least 29 proteins, including nonstructural proteins, accessory proteins, and four structural proteins: the spike (S), envelope (E), membrane (M), and nucleocapsid (N) proteins (50) (**Figure 2**). Their large genome favors coronaviruses to tolerate a wide range of ecological niches, and various hosts as well. As the viral tropism relies on viral and host proteins, the evolution of viral proteins plays an indispensable role in coronaviruses, from host recognition to genome replication and evasion of the host immune infrastructure. Among the structural proteins, the S protein is produced as a dimer or trimer. It performs two major functions: attachment to host cell receptors and facilitation of virion–host membrane fusion with host cell membranes (51). While the M protein functions in viral assembly and drives morphogenesis, while the E protein encapsulates viral RNA (52). Another multidomain N protein performs several crucial functions, including binding, compacting, and packaging the viral genome (53). A concise summary of the biological roles of SARS-CoV-2 nonstructural proteins is provided in **Supplementary Table 1**.

**Figure 3:**
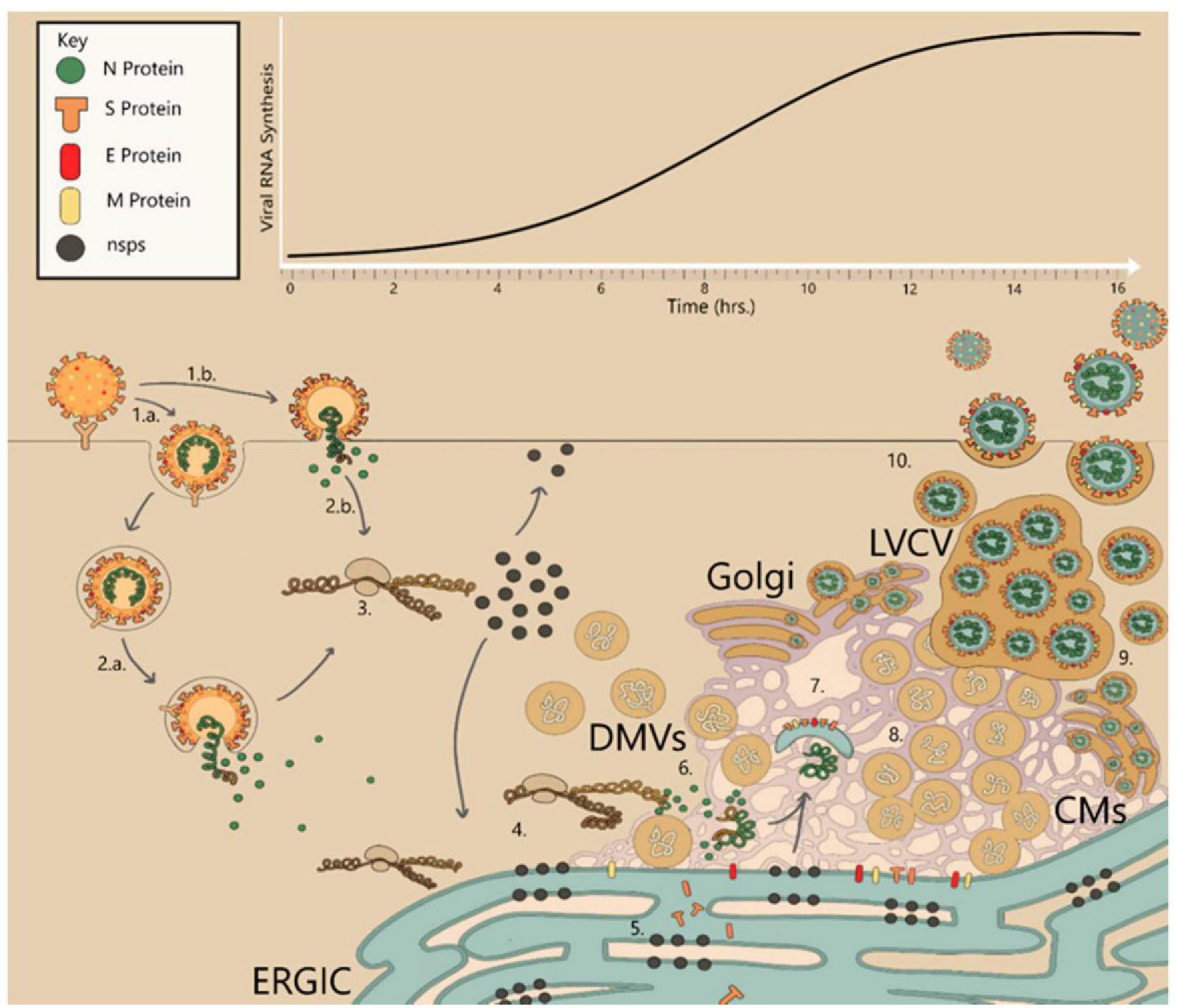
The coronavirus infection cycle inside the host cell. Figure reproduced from (54), an open access article distributed under the terms of the Creative Commons Attribution License (CC BY 4.0; https://creativecommons.org/licenses/by/4.0/). 1a) Endocytic entry of virus particles. 1b) Host cell recognition and attachment: immediate fusion at the surface of the cell upon attachment to a receptor protein. 2a) Endocytic fusion of viral and host membranes after endosomal maturation. 3) Uncoating and release of viral RNA; processing of replicase polyproteins by viral proteases to generate nsps. 4) Replication and transcription complex in the double-membrane vesicles (DMVs). 5) Translation of subgenomic RNA (sub-gRNA) into structural and accessory proteins. 6) Nucleoprotein complex formation. 7) Virion assembly; encapsulated RNA wrapped by lipid envelopes assembled by structural proteins. 8) Interconnected DMVs in the perinuclear space containing double-stranded RNA (dsRNA). 9) Mature CoV virions bud from ERGIC. 10) Release of virions via exocytosis.

The SARS-CoV-2 infection cycle requires host proteins (including a cohort of enzymes) and viral proteins. It mainly involves host cell recognition and entry into the host cytoplasm, viral genome replication and transcription, protein maturation, and release of viral particles (**Figure 3**). Viral gene expression and RNA synthesis are highly programmed and complex processes that occur in a coordinated spatiotemporal manner (**Figure 3**, **Supplementary Table 1**).

## 2 Effects of SARS-CoV-2 infection and inter-organ pathobiological links for progression of new-onset DM

### 2.1 SARS-CoV-2 viral entry and cell invasion

To shed light on the mechanistic links between viral tropism and pancreatic involvement, it is important to characterize pancreatic expression of ACE2, serine proteases, and other gateways for SARS-CoV-2 entry into different islet cell subtypes. In this section, we summarize expression profiles of the most common SARS-CoV-2 receptors in the pancreas and highlight gene and protein expression patterns associated with pancreatic β-cell pathophysiology.

#### 2.1.1 ACE2

Findings from RNA sequencing and immunohistochemistry have well established that ACE2 is expressed in many host tissues (55, 56). It is widely expressed in the nasopharynx, airways, lungs, gut epithelium, liver, pancreas, vascular endothelium, kidneys, adipose tissue, reticuloendothelial system, and central nervous system (27, 57–59).

ACE2 is expressed in human pancreatic β-cells (60) and pancreatic α-cells (61), and also in the pancreatic islet, ductal, and endothelial cells of non-human primates (NHPs) infected with SARS-CoV-2 (16, 62, 63).

Several experimental studies have shown prominent expression of ACE2 in pancreatic microvascular and ductal epithelium (64, 65). Despite some discrepancies in the data, increased ACE2 and TMPRSS2 expression has been reported in males and with increasing age (56), that may explain the disease severity among older men infected with SARS-CoV-2 (66). While some studies have reported the co-expression of TMPRSS2 and ACE2 (56), transcriptional profiling of primary human islet cells did not detect co-expression in single β-cells (60). Moreover, ACE2 and TMPRSS2 expression in pancreatic tissue remains controversial. For instance, one study reported absence of ACE2 and TMPRSS2 expression in β-cells of healthy and diabetic donors (60). However, a subset of human pancreatic ductal cells highly expressed ACE2 and TMPRSS2 in both diabetic and non-diabetic individuals (60, 67).

Limitations in methods, sex and ethnic background (68), and methods of sample preparation or preservation (69) could be possible reasons for these observed differences. Moreover, the presence of genetic variants or ACE2 and TMPRSS2 polymorphisms across different populations may contribute to these differences (70, 71).

Experimental studies in rodents showed increased expression of ACE2 in diabetes (72, 73). Particularly, increased ACE2 expression was observed in T2DM (68, 74). In line with these reports, as compared to healthy individuals, significantly higher ACE2 activity was found in diabetic and COVID-19 patients (75), suggesting that having diabetes may partly increase susceptibility to SARS-CoV-2 infection.

Overall, ACE2 expression may be regulated by different mechanisms, such as cytokine responses (76), ACE2 autoantibodies (77), host factors such as age and sex (68) and other regulatory elements such as growth factors (78, 79).

#### 2.1.2 TMPRSS2

The cellular serine protease TMPRSS2 is the key endopeptidase responsible for S protein (S2) priming, enabling viral entry into host cells (57). ACE2 comprises an arginine- and lysine-rich region, ^697^RTEVEKAIRMSRSRINDAFR^716^, which is essential for proteolysis by TMPRSS enzymes (80).

TMPRSS2 is commonly known as an androgen-responsive gene (81). Multiple RNA sequencing and immunohistochemistry studies, as well as RNA and protein data from the Human Protein Atlas, have documented TMPRSS2 expression in the pancreas, liver, lung, thyroid, gastrointestinal tract, kidney, salivary gland, and brain (82, 83). In androgen-sensitive tissues such as the prostate and testis, the expression of TMPRSS2 were found to be more significant (70), which may contribute to the increased vulnerability of males to SARS-CoV-2 infection. Higher TMPRSS2 levels have been observed in hyperglycemia and diabetes (84). A two-fold increase in TMPRSS2 expression in pancreatic β-cells of T2DM subjects compared with controls has also been reported (82).

Other transmembrane proteases, such as TMPRSS4, are also highly expressed in the stomach, various segments of the small intestine, esophagus, liver, and pancreas of SARS-CoV-2–infected individuals (16, 64).

#### 2.1.3 Neuropilin 1

NRP-1 is a cell-surface receptor involved in a wide array of molecular mechanisms, including angiogenesis, regulation of vascular permeability, and nervous system development (85). X-ray crystallography and biochemical studies by Daly et al. revealed that NRP-1 binds a polybasic Arg-Arg-Ala-Arg carboxyl-terminal sequence –a furin-cleaved substrate generated on the S1 subunit of the spike protein (86) –thereby promoting SARS-CoV-2 entry and contributing to cellular and organ tropism (87).

Several tissues express NRP-1. Notably, it is highly expressed in pancreatic β-cells rather than in α-cells (16, 65, 88). Studies have indicated a potential link between NRP-1 overexpression and the development of acute pancreatitis, potentially through promoting the release of proinflammatory cytokines (63, 89). Wu et al. demonstrated that pancreatic NRP-1 knockout prevented β-cell apoptosis (16). Additionally, a recent experimental study in mice revealed a critical role for NRP-1 in maintaining intraislet regulatory T-cell function and stability in autoimmune disease, thereby limiting diabetes onset (90).

#### 2.1.4 Furin

Furin proteases belong to the family of calcium-dependent proprotein or prohormone convertases (PCs) that are ubiquitously expressed in humans (91). The SARS-CoV-2 S protein contains a polybasic furin cleavage site, PRRAR (Proline–Arginine–Arginine–Alanine–Arginine), at the S1/S2 junction that facilitates increased S cleavage (92). Furin genetic variants or alleles can affect binding affinity for the viral S protein, potentially resulting in different degrees of infectivity (93). For instance, furin gene variant rs1981458 has been associated with COVID-19 severity (94).

*In vivo* studies show that knockout of the furin cleavage site attenuates SARS-CoV-2 pathogenesis (95, 96). Moreover, an imbalanced activity is also linked to pathologies such as infectious diseases, cancer, hypercholesterolemia, and atherosclerosis. Higher furin expression has been observed in diabetic patients compared with nondiabetic individuals, and elevated plasma furin concentrations may serve as a predictive factor for disease onset, progression, and premature mortality in diabetic patients (97). Importantly, furin is strongly associated with increased risk of diabetes, hypertension, and hyperlipidemia (98, 99). Moreover, *in vivo* studies demonstrate higher furin expression in pancreatic islets, and β-cell–specific furin knockout mice exhibit glucose intolerance due to smaller islets and reduced insulin content (100, 101). Furin maintains the growth of pancreatic β-cells and supports maturation of insulin secretory granules (102). These lines of evidence suggest that the possible mechanism of action of furin may operate via pancreatic β-cells. Thus, furin downregulation can reduce maturation of the insulin receptor and is linked to pancreatic β-cell dysfunction and insulin resistance.

SARS-CoV-2 viral entry receptors—including ACE2, TMPRSS2, furin, and NRP-1—are expressed in the lungs and multiple other tissues, such as the oral mucosa and salivary gland tissue (66, 103). The expressions can be cell-type-specific, and double positive expression (e.g., ACE2*TMPRSS2) has been observed in several cellular subsets of cell types (56). Multi-label immunofluorescence studies clearly demonstrate ACE2, NRP-1, and TMPRSS2 expression within β- and α-cells in autopsy and non-human primate samples (62, 63).

SARS-CoV-2 entry is not restricted to ACE2, TMPRSS2, NRP-1, or furin. For example, the endosomal proteases Cathepsin B/L can substitute for TMPRSS2 activity during S-protein priming (104). Furthermore, host receptors documented in COVID-19 individuals include dipeptidyl peptidase-4 (DPP4), transferrin receptor (TFRC), and extracellular high mobility group box 1 (HMBG1) (16, 65). Another host factor, placenta-associated protein (PLAC8), has emerged as an essential viral receptor, including in SARS-CoV-2 pancreatic infections (105, 106). Examination of postmortem materials from COVID-19 patients shows PLAC8 expression across various organs (105). Interestingly, evolving evidences show the diverse roles of PLAC8 in fundamental cellular processes such as proliferation, differentiation, and autophagy among others (107). It also influences AKT/mTOR, MEK/ERK, and Wnt/β-catenin signaling pathways (108).

### 2.2 Effect of SARS-CoV-2 on immune tolerance

A failure to maintain immune tolerance (i.e., the state in which the immune system is unresponsive to self-antigens) is the underlying cause leading to the development of autoimmune disorders, including T1DM. Thus, regulation of immune tolerance is critical to immune homeostasis (109). Regulatory immune cells, particularly CD4^+^CD25^+^FoxP3^+^ regulatory T cells (Treg cells), play a crucial role in maintaining and stabilizing immune tolerance via the production of powerful inhibitory cytokines (110). Of note, in the pancreatic islet niches, Tregs act as peacekeepers of immune tolerance to protect β-cells by inhibiting autoreactive β-cells and suppressing the differentiation and function of autoantigen-specific cytotoxic CD8^+^ T cells (111). Critically, viral infection can compromise immune self-tolerance. There is decades of evidence supporting the link between Epstein–Barr virus infection and multiple sclerosis. Similarly, compelling evidence suggests that SARS-CoV-2 compromises immune self-tolerance through mechanisms shared with other viruses, such as depleting Treg cell numbers or impairing their function via repression of FoxP3 expression (**i.e.,** a key transcription factor of Treg cells) (112). Importantly, loss of the protective function of CD4^+^CD25^+^FoxP3^+^ Treg cells has been tied to T1DM (111, 113). Notably, Anindya et al. demonstrated that SARS-CoV-2 infection leads to dysregulation of immune homeostasis, including compromised self-tolerance, diminished Treg function, and Treg instability, which represents a potential root of T1DM onset (114). In line with this, several autoimmune mechanisms—including molecular mimicry, epitope spreading, and bystander activation—have been hypothesized as potential mechanisms by which SARS-CoV-2 might trigger T1DM (115).

Another crucial point is the potential mechanistic link between virus-induced IFN signaling and T1DM. Although the link is complex, both conditions share a chronic inflammatory state. Studies have revealed impaired type I interferon (IFN-I) responses in the blood of severe and critical COVID-19 patients. This delayed or exhausted interferon production may contribute to desensitization or a refractory state in IFN signaling, promoting ongoing viral replication and hyperinflammation (116). Consequently, this exacerbated inflammatory response, accompanied by high levels of IL-6 and TNF-α, may induce insulin resistance and hyperglycemia. Similarly, elevated IFN-I signaling may trigger both the initiation and progression of autoimmune diabetes. It is strongly hypothesized that IFN-α and IFN-γ exert direct effects on β-cells, leading to β-cell toxicity. Moreover, virus-induced IFN-γ can directly cause insulin resistance (117).

Collectively, this evidence indicates that SARS-CoV-2 infection may act as an environmental trigger for T1DM by directly damaging β-cells and disrupting the protective role of Treg cells within the pancreatic islet niche. Therefore, virus-induced autoimmune responses, systemic inflammation, immunologic injury, exhaustion of pancreatic β-cells, and stress-induced hyperglycemia may contribute to the progression of new-onset T1DM and T2DM.

### 2.3 Effects on pancreas and other endocrine tissues (structural and functional abnormalities during pre- and post-SARS-CoV-2 infection)

To gain insights into the progression of T1DM and T2DM, it is important to understand the structural and functional abnormalities of pancreatic tissues before and after SARS-CoV-2 infection. Studies at the molecular level—including expression of antiapoptotic genes, endoplasmic reticulum stress–related genes, viral recognition pathways, and innate immune response genes in β-cells and α-cells—could also provide therapeutic strategies.

Mounting evidence shows that SARS-CoV-2 enters pancreatic cells and establishes infection, thereby associating with morphological, transcriptional, and functional changes of the pancreas. An early study by Yang et al. demonstrated SARS-CoV-2 infection of human pancreatic α and β cells (118). In this regard, pancreatic culture systems containing endocrine and exocrine cells, such as human induced pluripotent stem cell (iPSC)-derived cultures, have been widely used. Shaharuddin et al. used iPSC-derived pancreatic cultures to demonstrate deleterious effects of SARS-CoV-2 on normal molecular and cellular phenotypes. SARS-CoV-2 infection of the pancreas has also been confirmed in postmortem tissues from COVID-19 patients (119).

Pancreatic histopathology and immunofluorescence analyses revealed the presence of SARS-CoV-2 spike and nucleocapsid proteins in the islets and acinar epithelium of the acini (65, 105, 120). SARS-CoV-2 infection of islets causes both morphological and functional changes. Several studies reported increased numbers of insulin- and glucagon-positive cells, with a profound reduction in insulin-secreting granules in β-cells, suppressed insulin gene transcription, and impaired insulin secretion (16, 88, 120). Normal pancreatic cells exhibit ductal, acinar, or endocrine architecture, whereas SARS-CoV-2–infected cells show pancreatic tissue hyperplasticity, islet shrinkage, degeneration, and β-cell polyploidy. Mild lymphocytic infiltration of the endocrine and exocrine pancreas has been documented in some SARS-CoV-2–infected individuals with SARS-CoV-2 infection (65, 105). Such studies reported evidence supporting significant long-term morphological defects of the pancreas upon SARS-CoV-2 infection. For instance, histologic examination of pancreatic sections from postmortem tissues and infected pancreatic tissues showed thrombotic lesions, fibrosis, necroptotic cell death, immune cell infiltration, chronic pancreatitis, and focal acute pancreatitis (55, 65, 121). As demonstrated by the work of Steenblock et al., both intact islets and impaired islets were evident in COVID-19 patients without a previous diabetes diagnosis (65). In addition, examination of postmortem pancreatic tissue (from newly hyperglycemic patients with COVID-19 showed mild lymphocytic infiltration of pancreatic islets and pancreatic lymph nodes, presence of SARS-CoV-2–specific viral RNA, and several immature insulin granules (proinsulin) (122). Furthermore, crystalline morphology of β-cells, β-cell death, a substantial decrease in islet size, and a slight reduction in insulin expression have been observed (122). These findings highlight major structural and functional alterations—including β-cell degeneration, altered proinsulin processing, and β-cell hyperstimulation—among others that could eventually lead to disturbances in glucose homeostasis.

SARS-CoV-2 infection induces cellular trans-differentiation. For instance, while β-cells showed downregulated expression of insulin, higher expression of glucagon in α-cells and trypsin as a pancreatic acinar cell marker were observed upon SARS-CoV-2 infection (88). Similar findings by Wu et al. confirmed reduced pancreatic insulin levels and secretion, as well as β-cell apoptosis triggered by SARS-CoV-2 infection. Interestingly, phosphoproteomic pathway results showed similar apoptotic β-cell signaling between islets infected with SARS-CoV-2 and T1DM (16).

Cellular stress and increasing chemokine responses are hallmarks of SARS-CoV-2 infection. One notable mechanism through which SARS-CoV-2 perturbs key inflammatory responses is by hijacking the ribosomal machinery. For instance, as demonstrated in iPSC-derived pancreatic cultures, perturbed inflammatory responses—such as upregulation of the cytokine stromal cell–derived factor 1 (SDF-1) or CXC motif chemokine 12 (CXCL12)—were observed in the pancreas upon SARS-CoV-2 infection (119).

Metabolic stress is a critical factor that can lead to pancreatic β-cell dedifferentiation, thereby causing downregulation of ACE2 and other key proteins. For instance, high-fat diet (HFD)–induced insulin resistance and glucose intolerance are associated with decreased ACE2 expression. It is noteworthy that under HFD conditions, a higher percentage of dedifferentiated β-cells was detected in ACE2-knockout mice than in wild-type mice (123). Moreover, diet-induced obesity promotes delayed virus clearance, as demonstrated in HFD mouse models (124). Critical metabolic disorders have also been observed in adult nonhuman primates (NHPs) infected with SARS-CoV-2 (63). According to this study, major loss of β-cells and attenuated insulin expression were evident, associated with complex and severe pathological conditions such as islet amyloidosis and necrosis, activation of alpha-smooth muscle actin (α-SMA), and aggravated fibrosis with lower serum collagen. Furthermore, increased pancreatic inflammation and stress markers—intercellular adhesion molecule 1 (ICAM-1) and Ras GTPase–activating protein-binding protein 1 (G3BP1)—were observed (63). In line with previous findings, new-onset DM accompanied by pancreatic damage (e.g., generalized fibrosis with multiple vascular thrombi) was observed in SARS-CoV-2–infected NHPs (62).

Patients without preexisting diabetes can also develop persistent hyperglycemia as a result of viral-mediated pancreatic damage (125, 126). It is also possible that even without developing new-onset diabetes, immune cell infiltration and necroptotic cell death may lead to varying degrees of metabolic dysfunction due to SARS-CoV-2–infected pancreatic β-cells (65).

Genes such as Synaptotagmin 4 (SYT4), Per-Arnt-Sim (PAS) domain-containing protein kinase (PASK), Phospholipase C X domain 3 (PLCXD3), and Peroxisomal biogenesis factor 6 (PEX6) are linked to β-cell physiology or DM (127–130). Interestingly, an elegant work by Müller et al. revealed significant dysregulation of these genes in uninfected and infected cultured human islets. SARS-CoV-2 infection resulted in downregulation of SYT4, PASK, PLCXD3, and PEX6, while several interferon (IFN)–stimulated genes (ISGs), such as IFITMs, OAS2, IFI27, and ISG15, were upregulated (120). Similarly, in other tissues SARS-CoV-2 elicits higher ISG transcript levels. For example, in an organ-specific manner (ovary, pancreas, and thyroid), downregulation of endocrine-specific genes such as IAPP, HSD3B2, INS, LEP, FOXE1, TSHR, and CRYGD has been identified in COVID-19 patients (131). Many molecules essential to islet function—including CD36 (regulates β-cell response to hyperglycemia and glucolipotoxicity), insulin receptor substrates IRS1 and IRS2, glucose transporters GLUT2, PPARG, and pancreatic and duodenal homeobox 1 (PDX1)—play key roles in the pathogenesis of diabetes (132, 133). Of note, SARS-CoV-2 infection provokes dysregulation of CD36, GLUT2, IRS1, IRS2, PDX1, and PPARG expression in the pancreas (134).

**Figure 4:**
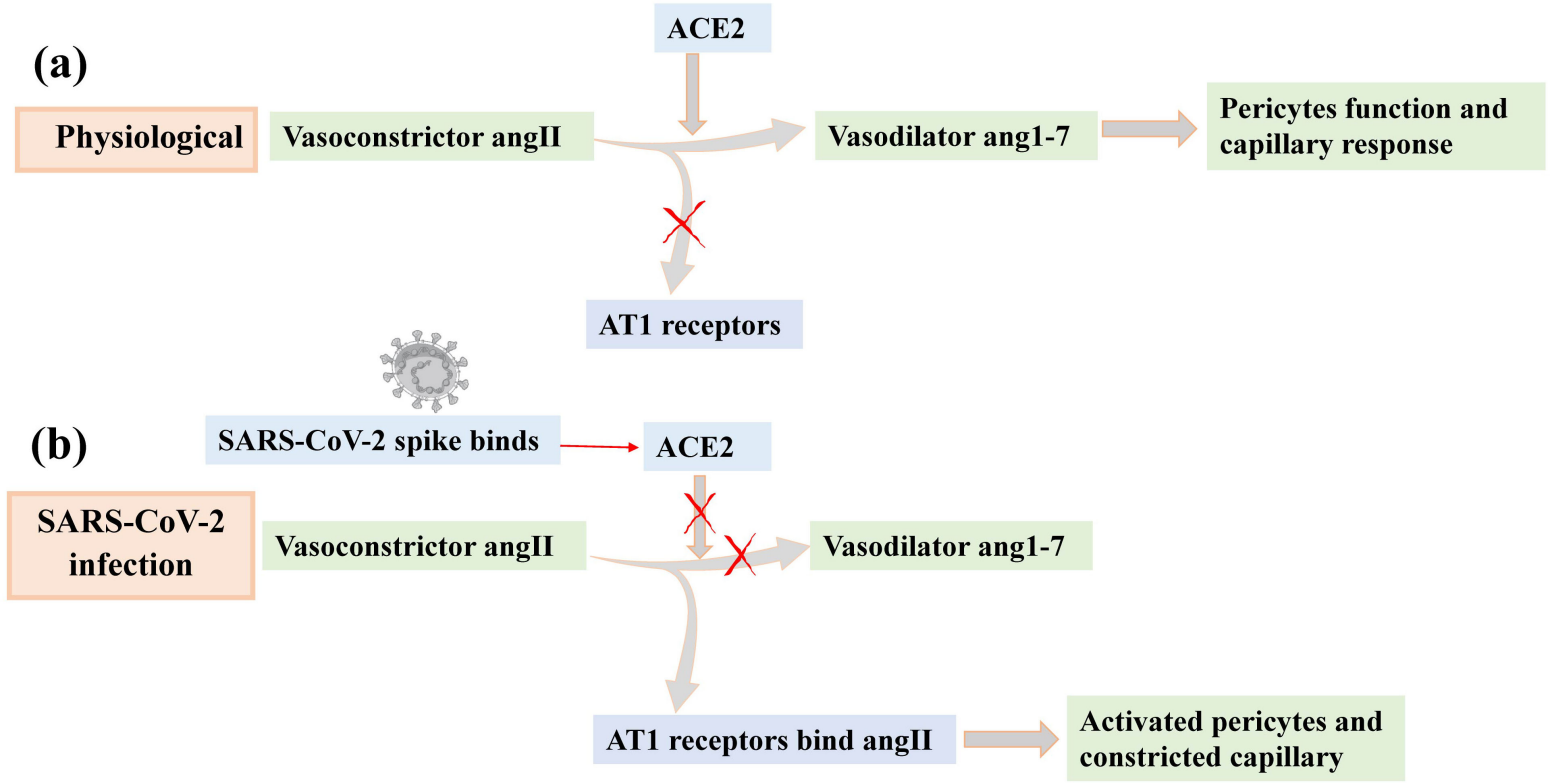
Schematic illustration of impairment of islet pericyte function upon SARS-CoV-2 infection. Different components of the local renin–angiotensin system (RAS), such as ACE2 and angiotensin type 1 receptors (AT1 receptors), are expressed in the pericytes of the pancreatic islets (139). **(a)** Under physiologic conditions, ACE2 degrades the vasoconstrictor Ang II into the vasodilator Ang 1–7, preventing Ang II from activating AT1 receptors, thereby maintaining pericyte function and capillary reactivity. **(b)** During SARS-CoV-2 infection, ACE2 binds to spike protein, becomes internalized, and no longer degrades Ang II into Ang 1–7. This results in an increase in endogenous levels of Ang II in the pancreas. Ang II then binds to AT1 receptors in pericytes, activating them and compromising vasomotor responses. Figure produced from (139).

Given that destruction of β-cells is relevant to both T1DM and T2DM, it is important to understand different SARS-CoV-2 targeted cell types involved in β-cells physiology and function. For example, *in vitro* infection of human pancreatic islets revealed that SARS-CoV-2 targets adventitial cells and pericytes (135). Pericytes are mural cells located within the basement membrane of blood microvessels that supply trophic factors crucial for maturation and proper function of β-cell (136) and regulate islet capillary diameter and blood flow (137, 138). Pericytes in the pancreas and other organs, such as the central nervous system and heart, express ACE2 (61). Examination of living pancreas slices from nondiabetic individuals showed that SARS-CoV-2 recombinant spike S1 proteins impair ACE2 activity and induce islet pericyte and microvascular dysfunction (139). This study offers new insights into molecular mechanisms such as vascular dysfunction, activation of pericytes, and islet capillary constriction upon SARS-CoV-2 infection (139) (**Figure 4**). This confirms the diabetogenic actions of SARS-CoV-2 through pancreatic islet pericyte dysfunction and disturbance of glucose homeostasis.

SARS-CoV-2 profoundly affects endocrine organs/glands, such as the hypothalamo–pituitary–adrenal axis, thereby disturbing glucose metabolism. It can worsen glycemic control in individuals. Thus, new-onset hyperglycemia, also termed *“stress hyperglycemia*,” observed in patients with severe COVID-19 illness could be significantly influenced by the stress response. The increasing levels of stress hormones such as catecholamines and cortisol can provoke insulin resistance and promote blood glucose elevation, consequently leading to poor outcomes in pre-diabetic individuals (140).

Follow-up studies at the community level would help determine the extent to which insulin resistance and β-cell dysfunction contribute to new-onset diabetes. For instance, one recent observational follow-up study reported a greater risk of new-onset DM due to exacerbated insulin resistance rather than β-cell dysfunction (141). Furthermore, studies focusing on homeostatic maintenance and turnover, regenerative mechanisms, islet maturation, and cell type specification may help dissect the underlying molecular mechanisms and provide new avenues for understanding SARS-CoV-2–associated new-onset diabetes.

### 2.4 SARS-CoV-2 effects on the gastro-intestinal tract

**Figure 5:**
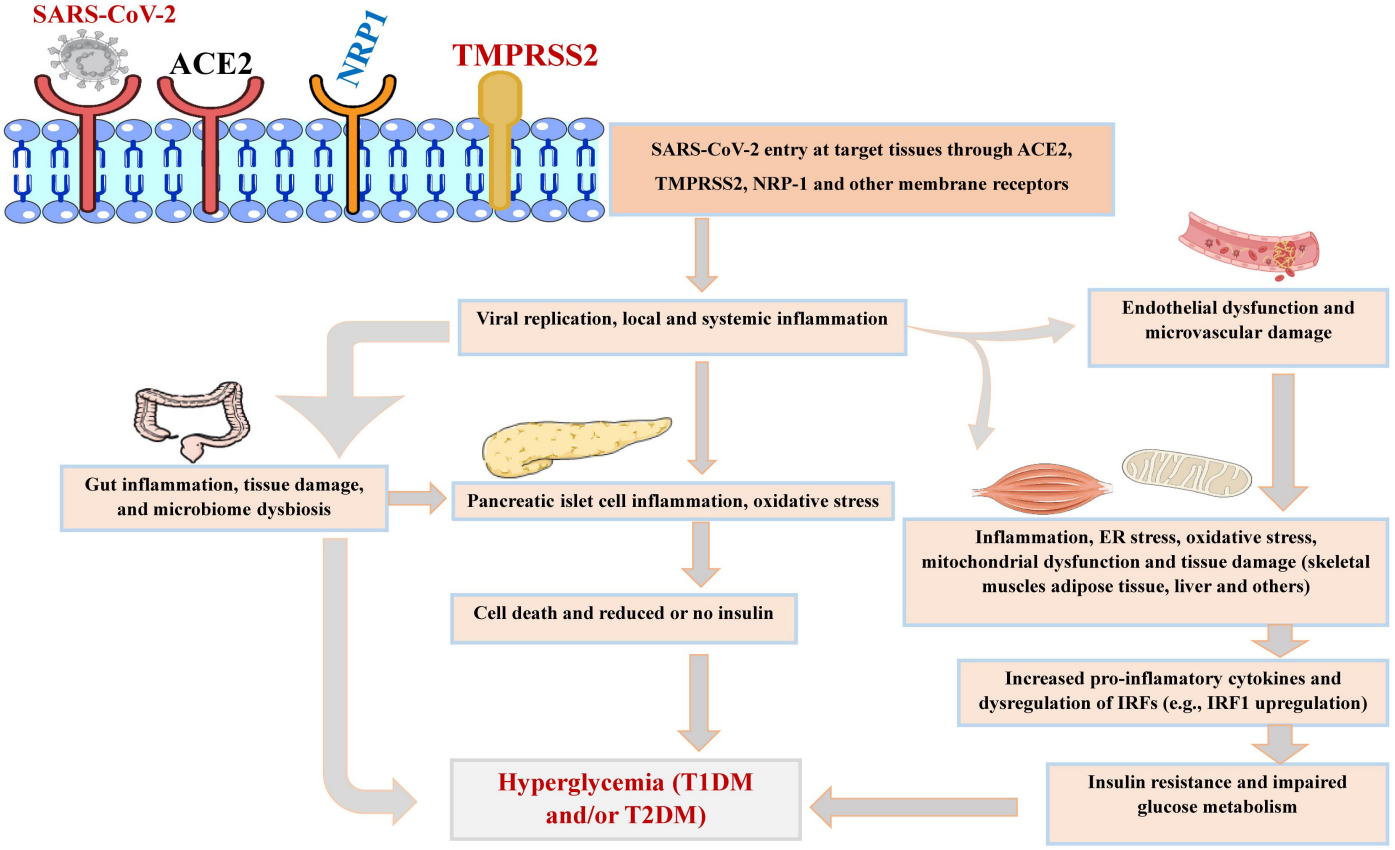
Diagram depicting SARS-CoV-2 viral entry (through ACE2, TMPRSS2, NRP1, and other alternative receptors) into host cells and related effects on multiple organs and tissues. SARS-CoV-2 affects different pathways in several tissues, leading to potential metabolic disturbances that could elicit hyperglycemia and the development of T1DM and/or T2DM. ACE2, angiotensin-converting enzyme 2; IRFs, interferon regulatory factors; ER stress, endoplasmic reticulum stress; NRP1, neuropilin-1; TMPRSS2, transmembrane serine protease 2. Parts of the figure were drawn and adapted from Servier Medical Art (http://smart.servier.com), licensed under CC BY 4.0 (https://creativecommons.org/licenses/by/4.0/).

The expression of ACE2 can be upregulated by inflammatory cytokines following SARS-CoV-2 infection in airway epithelium (142). The virus itself may also upregulate ACE2 expression, suggesting a positive feed-forward effect that enhances viral infection (143), potentially leading to increased inflammation and multi-organ damage in tissues such as the airways, intestines, pancreas, liver, and vasculature (**Figure 5**).

*In vitro*, SARS-CoV-2 infection induces expression of proinflammatory genes such as CCL2, CCL3, CCL5, CCL10, IL-1β, and IL-6 in human small intestinal epithelial cells derived from pluripotent stem cells (144). The inflammatory response in the gut may increase barrier damage and contribute to the gastrointestinal symptoms observed following infection.

Notably, a growing body of literature highlights the role of gut microbiota in various metabolic diseases. It is well established that SARS-CoV-2 infection and viral entry into enterocytes lead to gut microbiome dysbiosis, which can disrupt gut barrier integrity and promote inflammation, along with altering metabolite levels that may be linked to new-onset DM (144, 145). Such gastrointestinal damage may also result in malabsorption of vital nutrients, including vitamins, which play indispensable roles in metabolism.

Persistent gut damage caused by SARS-CoV-2 infection has been shown to induce long-term dysregulation of tryptophan (Trp) absorption from the intestine (146) potentially contributing to the drastic decrease in plasma Trp levels in long COVID patients (147). In addition, viral infection and type I IFN–driven inflammation also cause serotonin reduction through decreased intestinal absorption of the serotonin precursor Trp, platelet hyperactivation, and thrombocytopenia, all of which affect serotonin storage. Notably, serotonin reduction has been associated with PASC (146). Another study indicated decreased peroxisome biogenesis and enhanced peroxisome degradation as a result of increased IFN levels following SARS-CoV-2 infection and inflammation, revealing peroxisomes as essential regulators of macrophage-mediated lung inflammation resolution and tissue regeneration (148). Hence, since peroxisomes play an important roles in lipid metabolism and immunometabolism, dysregulation of their function can disrupt lipid balance, resulting in considerable cellular stress and immune dysfunction.

However, it is important to note that gut microbiota may be associated with T2DM either positively or negatively. For instance, a systematic review reported that the genera *Bifidobacterium, Bacteroides, Faecalibacterium, Akkermansia*, and *Roseburia* were negatively associated with T2DM (149). A meta-analysis in antibiotic-naïve COVID-19 patients showed reductions in beneficial (symbiotic) bacteria and increased levels of opportunistic pathogens, including *Actinomyces viscosus, Clostridium hathewayi*, and *Bacteroides nordii*, compared with controls (150). Recent findings indicate that gut microbiota alterations—due to various causes including SARS-CoV-2 infection—affect the metabolism of peptides and amino acids (e.g., tryptophan, phenylalanine, glutamate, citrulline), short-chain fatty acids (SCFAs), xenobiotics, and other metabolites, which may negatively influence infection rates, inflammation, and glycometabolic abnormalities, including T2DM (150, 151). Additionally, although not COVID-19–specific, microbiota changes in *Clostridium* species have been linked to predicted plasma metabolites involved in phenylalanine metabolism—such as phenylacetate, phenylacetylglutamate, and phenylacetylglutamine—which correlate with heightened cardiovascular risk and T2DM (152). A recent metabolomics study of 1,167 individuals identified blood metabolites, including lipid- and amino acid–related metabolites associated with impaired glucose control, with most DM patients showing altered gut microbiome interactions involving *Hominifimenecus microfluidus* and *Blautia wexlerae* via Hippurate (151).

Notably, long COVID has been linked to gut microbiome dysbiosis, which contributes to insulin resistance and T2DM. These findings highlight the critical effect of SARS-CoV-2 infection on the gut microbiota, leading to a decrease in both the total number of organisms and the variety of species present. Such microbiome dysbiosis and microbial translocation may contribute to metabolic derangements and the progression of new-onset diabetes.

### 2.5 SARS-CoV-2 effects on adipose tissue, skeletal muscles, and vasculature

A study conducted on glucose-utilizing tissues such as adipose tissue showed higher ACE2 expression than in lung tissue, which could be linked with ACE2-mediated viral replication and pathogenesis, including oxidative stress and inflammation in such tissues as well (58).

Researchers detected SARS-CoV-2 RNA in adipose tissue specimens (visceral, epicardial, pericardial, and subcutaneous) from autopsy samples of deceased patients with COVID-19 undergoing cardiothoracic surgery, along with inflammatory infiltrates, suggesting that the virus also infects inflammatory adipose tissue–resident macrophages and adipocytes (153). Several other research groups also reported detection of SARS-CoV-2 RNA in abdominal subcutaneous adipose tissue specimens, including 13 of 23 COVID-19 autopsy cases (154), 28 of 59 cases of human adipocytes (155) and experimental infection of adipose tissue in the hamster model of SARS-CoV-2 infection (156).

Bulk RNA-seq analysis revealed that lungs and adipose tissue specimens had various immune cells and inflammatory markers (e.g., IL-6) upregulated in COVID-19–infected obese K18 mice (n=4) as compared with COVID-19–infected normal or non-obese (n=4) control mice (157). The data from this model study support a link between inflammation and increased adipose mass or obesity-related COVID-19 infection, which in turn could be associated with glycometabolic disturbances such as insulin resistance. Interestingly, transcriptome analysis conducted on lung and adipose tissue specimens obtained from deceased COVID-19 patients also found that obese patients (n=3) had increased expression of genes and activation of inflammation-related pathways as compared with normal-weight COVID-19 patient tissues (n=2) (157).

A longitudinal study revealed that SARS-CoV-2 infection provokes systemic metabolic abnormalities, mitochondrial dysfunction of skeletal muscle, and abnormal skeletal muscle response to exercise (including exercise-induced myopathy and tissue infiltration of amyloid-containing deposits and leukocytes) (28).

In individuals with severe COVID-19 illness, long-lasting liver damage is observed, and ACE2 expression in cholangiocytes has been hypothesized as a potential cause of liver damage (158, 159). NRP-1 expression in various body tissues or cell types, including liver-resident cells such as sinusoidal endothelial cells and hepatic stellate cells, may also facilitate SARS-CoV-2 infection, thereby increasing liver damage and decreasing hepatic function (160).

Besides pulmonary and other complications, endothelial injury has been established as a primary finding in patients infected with SARS-CoV-2, and the vasculature has been clinically observed as one of the main trans-organ systems affected by SARS-CoV-2 infection. A postmortem histopathological study revealed viral inclusions in apoptotic endothelial cells, infiltration of inflammatory cells, microvascular endothelitis, and vascular complications (161). Furthermore, SARS-CoV-2 infection leads to complex and multifactorial endothelial cell activation, progressive loss of antithrombotic factors, and promotion of local pro-angiogenesis (162).

## 3 Incidence and molecular view of new-onset DM (T1DM and T2DM) following SARS-CoV-2 infection

It is clear that pre-existing diabetes and/or other chronic conditions (including obesity) have worsening effects on COVID-19 disease outcomes, showing strong associations with morbidity and mortality (142, 163). Although many studies conducted in adults infected with COVID-19 showed an increased risk of diabetes and other metabolic abnormalities (5, 15) (**Table 1**), causal relationship studies linking acute or long COVID-19 with DM or other chronic conditions are generally limited by short follow-up duration and/or other limitations.

**Table 1:** Some of the studies conducted and study outcomes showing risk of new-onset DM following SARS-CoV-2 infection.

Study design/setting, country	Tissue/organ/specimen	Study source (population)	Study outcomes (risk/incidence of DM)	Ref.
Retrospective cohort study (1 year follow-up), USA	Blood	Human adults (n= 181, 280 with COVID-19 and contemporary controls, n= 4,118, 441)	Increased risk of type 2 DM and antihyperglycemic use in those with COVID-19 (as compared with controls)	((24)
Retrospective cohort study, USA	Blood	Human adults (n= 353, 164 with COVID-19 and controls, n=1, 640, 776 controls	Increased risk of new onset type 1 DM and type 2 DM in those with COVID-19.	(15)
Retrospective cohort study, USA	Blood	Human, children (aged <18 years, n = 2.5 million)	Higher risk of new-onset diabetes (type 1 DM) in those with COVID-19	(14)
Retrospective cohort study, USA	Blood	Children and adolescents (age:10–19 years, n =613, 602 patients, n= 306, 801 with other respiratory infections	Higher risk of an incident diagnosis of Type1 DM & Type 2 DM	(164)
Observational follow-up study (base-line up to 6 months), India	Blood	Human adults (1,301 total subjects; n=855 COVID-19 patients and n=455 diabetic patients without COVID-19)	An incidence of 8.5% (n= 73) of new onset-DM (type 2 DM), 19% (n=163) with history of preexisting DM, and 72.5% (n=619) with euglycemia was found among 855 COVID-19 patients	(141)
Retrospective study, China	Blood	Human elderly patients with COVID-19 (total subjects= 453 patients, median age: 61 (IQR 49, 68) years)	Incidence of 21% (n=94 patients) new onset-DM, 21.6% with history of preexisting DM, and 28.4% with dysglycemia	(165)
Systematic review and meta-analysis, China/Italy/US	Blood	Human adults (total= 3,711 COVID-19 patients, age range: 47 and 64.9 years, gender: 53.3–80.0% male)	492 patients had new-onset DM (pooled prevalence of new-onset DM as 14.4% )	(166)

A large database study among adults in the United States conducted on a cohort of over 180 000 participants who had COVID-19 and a contemporary control group (over 118 000 individuals) with no DM before COVID-19 diagnosis indicated an increased risk of T2DM and antihyperglycemic use in both non-hospitalized and hospitalized individuals at 1-year follow-up after COVID-19 diagnosis (24). In other two large database studies conducted on adults in US, subjects with COVID-19 had an increased risk of new T1DM and T2DM (15).

Although the proportion of T1DM versus T2DM was not specified, analysis of two large databases of more than 2.5 million children (<18 years) demonstrated that children with COVID-19 showed an increased risk of new-onset DM compared with those without COVID-19 (14).

An increased risk of new-onset T2DM was also reported within 6 months following a COVID-19 diagnosis, compared with other respiratory infections, in a cohort of more than 600,000 patients aged 10–19 years (164). In another hospital-based observational follow-up study (up to 6 months) among 855 COVID-19 patients, 19% (n=163) had a history of preexisting DM, 72.5% (n=619) had euglycemia (neither preexisting DM nor new-onset hyperglycemia), and 8.5% (n=73) developed new-onset T2DM following COVID-19 infection (141). A retrospective study from China among hospitalized COVID-19 patients (mainly elderly) reported that 21.6% had a history of DM, 21% were newly diagnosed with DM, and 28.4% had dysglycemia (165). A systematic review and meta-analysis of eight studies (n=3711 patients) reported 492 newly diagnosed DM patients and a pooled prevalence of 14.4% for new-onset DM (166).

Physiologically, ACE2 is involved in the renin–angiotensin–aldosterone system (RAS), which plays diverse roles in regulating vascular function, blood pressure, inflammation, electrolyte balance, immune activity, and other homeostatic processes. In the RAS pathway, renin cleaves angiotensinogen to angiotensin I (Ang I), which is then cleaved by ACE to angiotensin II (Ang II), mainly in the lungs. Ang II binds to the vasoconstriction-mediating type 1 receptor (AT1R), leading to increased aldosterone production, vasoconstriction, sympathetic stimulation, oxidative stress, inflammation, and profibrotic and proliferative characteristics (167, 168). Ang II also binds the vasodilation-mediating type 2 receptor (AT2R), which counteracts detrimental RAS effects by promoting vasodilation, anti-inflammatory, anti-fibrotic, and anti-proliferative activities. ACE2, with its ubiquitous expression across various organ systems (the lungs, kidneys, GI tract, reproductive organs, and others), can also reduce the negative effects of Ang II through several mechanisms. For example, it can cleave Ang I to Ang1–9 or Ang II to Ang1–7, which mediate counter-regulatory effects against most of the deleterious actions of the ACE/Ang II/AT1R axis (anti-proliferative, anti-hypertensive, anti-inflammatory, and metabolic effects), especially in pathological conditions, via the proto-oncogene G-protein–coupled receptor called Mas receptor (MasR) (169, 170).

The data showing the variations of ACE2 expression (upregulation vs. downregulation) and RAS activity following SARS-CoV-2 entry have not been consistent or clear yet (171). Variations in RAS activity were noted during the various stages of SARS-CoV-2 infection, and evidence showed increased activation of the system in the early stages and then decreased activation in subsequent stages. Importantly, pathological conditions during SARS-CoV-2 infection may favor Ang II–driven inflammation through the production of IL-6, TNF-α, and other inflammatory cytokines in several host tissues and organs (159). Likewise, several factors—including cytokine response, age, sex, and the use of ACE inhibitor drugs (which can lead to increased ACE2 formation)—may be associated with ACE2 expression and RAS activity. The physiological balance of the RAS system, especially the ACE/ACE2 axis, which determines the subsequent products formed and their effects, appears to be dysregulated by SARS-CoV-2 viral infection (143, 172). Thus, once RAS balance is perturbed (pathological RAS) in COVID-19, it could lead to widespread dysfunction and damage in various target tissues and organs, including cascades of inflammation, immune system dysregulation, oxidative stress, vasoconstriction, disruption of fibrinolytic balance, and/or other metabolic abnormalities (58, 173, 174).

Moreover, studies showed SARS-CoV-2–associated skeletal muscle damage, including mitochondrial dysfunction and myopathic inflammatory responses, along with altered systemic metabolic profiles. Such PASC-associated structural and functional changes—particularly in the two major glucose-utilizing tissues, adipose tissue and skeletal muscle—could lead to alterations in glucose metabolism and progression of new-onset DM (58). It has also been noted that acute respiratory viral infections can enhance IFN production and promote insulin resistance in human skeletal muscles (117). Vascular complications in long COVID, involving endothelial dysfunction driven by heightened inflammatory responses, are also associated with increased risk of metabolic disorders such as new-onset DM (175, 176). It is also noteworthy that the formation and release of neutrophil extracellular traps (NETs), known as NETosis, is involved in innate immune responses during microbial infection. Persistent SARS-CoV-2 infection, in which NETosis is involved, creates a mechanistic link between long COVID and diabetes (177).

A multitude of inter-organ damages—including oxidative stress, inflammation, and mitochondrial and endothelial dysfunction—was reported by Shin et al., demonstrating the effects of SARS-CoV-2 on glucose metabolism through impaired insulin signaling pathways in human and animal tissues, including lungs, liver, adipose tissue, and pancreatic cells/tissues (178). In addition, impairment of several insulin/IGF signaling pathway genes—relevant to insulin resistance and compromised glucose metabolism—was attributed to increased interferon regulatory factor 1 (IRF1) expression and its observed activity or effect (178). Intriguingly, insulin resistance and impaired glucose metabolism leading to new-onset T2DM have been associated with IRF1 overexpression in PASC.

IRF1 is known to be one of nine IRFs involved in immune responses (primarily innate), immune cell development, and oncogenesis. IRF1 has constitutive expression and is also inducible in various mammalian cell types, with localization mainly in the nucleus and partly in the cytoplasm. Among other IRFs involved in immune responses, IRF1 stimulates expression of IFN-inducible genes (such as IFN-α) and proteins with diverse immune functions, including GBP, Caspase-1, Cox-2, CIITA, TAP1, LMP2, IFN-β, iNOS, IL-12p35, and IL-12p40. Therefore, besides an altered RAS pathway, increased expression and activity of IRF1 following SARS-CoV-2 infection (as in oncogenesis or other virus-induced infections) could serve as a molecular cross-talk mechanism for various shared inter-organ damages. Impaired glucose homeostasis in several tissues/organs, as described above through reduced expression of insulin signaling pathways (IRS1, PI3K, AKT, mTOR, MAPK cascade), could be mediated through IRF1 overexpression in PASC (178).

Generally, growing evidence shows that SARS-CoV-2 infection can result in RAS imbalance in various target organs/tissues (143) which may lead to negative effects in COVID-19 patients, including immune system deregulation, increased inflammation, and multi-organ damage affecting the lungs, pancreas, liver, vasculature, intestines, skeletal muscle, adipose tissue, kidneys, nervous system, and others (58, 173, 174). Eventually, such local and systemic pathophysiologic changes could be linked to metabolic disturbances such as progression of new-onset diabetes (T1DM and T2DM) and other conditions (178, 179).

The systemic inter-organ damages associated with altered RAS pathways could mechanistically contribute to impaired glucose metabolism through increased pro-inflammatory cytokines (154, 159) which in turn can act synergistically with immune dysregulation of IRF1 (or other IRFs such as IRF3, 7, and 9) following SARS-CoV-2 infection, further promoting inflammatory pathways (such as immune cell infiltration and tissue damage), insulin resistance, and persistent hyperglycemia (178). Overall, impaired glucose control in PASC may develop either through low insulin secretion by the pancreas (T1DM progression) or impaired glucose metabolism and insulin resistance (T2DM progression) in major target tissues (adipose tissue, skeletal muscle, and liver) due to downregulation of insulin signaling pathways (178). This, in turn, leads to reduced GLUT4-dependent glucose uptake, reduced intracellular glycogen storage, increased hepatic glucose production (versus reduced glucose utilization by tissues), and/or reduced transcriptional roles of insulin (180, 181), as summarized in **Figure 5** and **Table 2**.

**Table 2:** Summary of studies conducted and study outcomes showing the potential cellular/molecular mechanistic pathways for new-onset DM progression following SARS-CoV-2 infection.

Study design/setting, country	Tissue/ organ/specimen	Study source (population)	Pathophysiology markers (cell/tissue damage and molecular pathways)	Ref.
Clinical (*in vitro*)	Lung cultured cells	Human		(57)
Clinical *ex vivo* (immunohistochemistry assay)	Lung tissues and GI tract tissues	Human (two patients with non-small cell lung cancer and concurrent COVID-19	• TMPRSS4 was over expressed in lungs and other tissues (esophagus, stomach, small intestine, jejunum, ileum, colon, liver and pancreas)	(27)
Clinical (*ex vivo* and *in-vivo)*	Pancreatic islets	Human (13 non-COVID-19 pancreatic islet donors for ex vivo and 9 subjects who died from COVID-19 for *in vitro*)	• ACE2 TMPRSS2, NRP1, are expression in β-ells (the highly expressed NRP-1 receptor is critical for viral entry resulting in B-cell damage and decrease in insulin secretion)	(16)
Clinical (*in vitro)*	SARS-CoV-2 infected pancreatic islets	Human	• Largely non-cytopathic modest cellular perturbations and inflammatory effects	(135)
Clinical *ex vivo* (Gene expression database analysis)	Adipose and lung tissue culture	Human	• ACE2 expression (in SARS-CoV-2 infection) in adipose tissue was higher than that in lung tissue	(58)
Clinical (*ex vivo)*	Adipose tissue and other tissues (deceased specimens)	Human (10 adult humans infected with SARS-CoV-2 undergoing cardiothoracic surgery)	• Virus replication in patient tissues • Inflammatory responses in the adipose tissues and macrophages • ACE2 was not detected or expressed in adipocytes	(182)
Clinical (*ex vivo)*	Adipose tissue (abdominal adipose tissue from deceased subjects)	Human adults (n=23 COVID-19 patients and n=12 COVID-19 negative controls)	• Virus infection/replication in adipose tissue (13 SARS-CoV-2 positives out of 23, i.e. 56% of patients) • Inflammatory responses (leukocyte infiltration and upregulation of IFN-α pathway • ACE2 positive expression in the adipose tissues	(154)
Clinical *ex vivo* and experimental *in vivo* (transcriptome analysis)	Adipose tissue and lung tissue	• Human (2 infected with COVID-19) • 18 Mice model (obese &non-obese/ normal controls)	• Increased expression of genes and the activation of pathways associated with inflammation (immune cells and IL-6) as compared to normal-controls	(157)
Cohort study (metabolome profiling), Sweden	Blood and fecal samples	Human (total= 1,167; discovery cohort, *n* = 697, validation cohort, *n* = 470)	• Impaired glucose control (T2DM) in those with altered gut microbiomes (about 54.2% out of identified abnormal blood metabolome profiles were lipid-related and 20.3% as amino-acid-related)	(151)
Clinical cohort study (*in vivo* and *ex vivo*), Netherlands	Blood & skeletal muscle biopsy	Human (25 healthy controls and 21 long COVID-19)	SARS-CoV-2 infection leading to: • Mitochondrial dysfunction, myopathy and tissue infiltration of amyloid-containing deposits in skeletal muscle biopsies. • Systemic metabolic abnormalities	(28)
Clinical and experimental (transcriptome analysis)		• Human (public datasets of 2 SARS-CoV-2 infected cells/tissues (lungs, liver, adipose tissue, pancreas, blood) • Mice tissues/cells (ACE2 transgenic mice models infected with SARS-CoV-2)	• SARS-CoV-2 infection impaired insulin/IGF signaling pathway genes (IRS, PI3K, AKT, mTOR, and MAPK) in lungs, liver, adipose tissue, and pancreatic cells through increased interferon regulatory factor 1 (IRF1)	(178)

In addition to the observed increased risks and progression of new-onset diabetes, it could also be possible that people with COVID-19 and its associated disorders may have differentially experienced some broader factors (genetic, environmental, and other susceptibility factors such as social or economic) following the pandemic.

## 4 SARS-CoV-2 RNA in PASC reservoir sites such as the pancreas and other tissues

Despite no precise localization, it is now well understood that SARS-CoV-2 persists in several tissues for months to years (**Table 3**). The presence of viral RNA or fragments could directly damage different islets of the pancreas, contributing to insulin resistance and β-cell dysfunction. For instance, a prospective cohort study in non-diabetic individuals showed development of long COVID syndrome and insulin resistance in a large number of individuals (191). Therefore, it is of great importance to understand SARS-CoV-2 RNA transcription, translation, replication, and whether it is infectious in PASC reservoir sites.Another central question is the nature of viral RNA association with the development of long COVID symptoms. These issues would be addressed by detecting viral RNA from recovered SARS-CoV-2 patients. For instance, multiple studies reported higher levels of viral RNA in individuals with consistent PASC symptoms compared with fully recovered COVID patients (192, 193).

**Table 3:** Summary of the persistence of SARS-CoV-2 RNA, and viral proteins in human/non-human PASC reservoir tissues and its potential association with progression of diabetes mellitus/related metabolic changes.

Ref.	Samples/location	Methods	SARS-CoV-2 RNA/Genes expression, viral proteins or fragments (Time of detection)	Associated effect on progression of DM/ other metabolic complications
	Human tissue (biopsy)			
(183)	Gut lamina propria	• Whole-body positron emission tomography imaging with a tracer	• Spike RNA after 2 years post-infection	• There is no available information, however the observed persistent T cell activation, and inflammation could be linked to a range of metabolic complications such as DM. • Persistent T Cell activation in several tissues such as gut and spinal cord was found associated with long COVID symptoms.
(44)	Tissue biopsy from residual (tissue and blood samples); brain, blood vessel, liver, kidney, stomach, lung, breast, intestine, skin, and thyroid.	• Digital droplet PCR, RNA *in-situ* hybridization, immunofluorescence, immunohistochemistry	• Viral RNA was detected in 30% (16/53) solid tissue samples, 27% (38/141), and 11% (7/66) collected at 1, 2, 4 months, respectively. • Viral RNA detected in brain, blood vessel, liver, kidney, stomach, lung, breast, intestine, skin, and thyroid. • sgRNA detected	• Though the study does not mention whether it is before or post SARS-CoV-2 infection: 11% (8/72) individuals with long COVID had diabetes, and 9% (13/141) individuals without long COVID had diabetes.
(184)	Appendix, breast tissues and skin of 2 patients with long COVID symptoms	• RNAscope *in situ* hybridization • Multiplex immunohistochemistry	• Nucleocapsid protein detected in all the three tissues after 163 and 426 days of symptom onset • Nucleocapsid colocalized with macrophage marker CD68 • Viral RNA and spike protein detected •	• There is no available data reported on the DM, however, the presence of viral RNA and protein in gastrointestinal tract may contribute to gut dysbiosis and thereby, could contribute to metabolic related changes.
(185)	Stomach and gallbladder tissues	• Immunohistochemistry	• Nucleocapsid protein detected after 274 days of initial infection	• Diabetes, and dyslipidemia were reported as associated medical conditions
(186)	Liver biopsies (postmortem), and primary hepatocytes	• RT-qPCR	• Replicating viruses detected in human hepatocytes • NSP-16 were detected in liver biopsies	• SARS-CoV-2 infection independently induced hyperglycemia, irrespective of diabetic history and β cell function. • It also caused induction of phosphoenolpyruvate Carboxykinase (PEPCK) activity and gluconeogenesis.
	Human tissue (autopsy)			
(131)	Autoptic specimens of adrenals, pancreas, ovary, thyroid, and white adipose tissue	• RT-PCR	• Endocrine-specific genes such as HSD3B2, INS, IAPP, TSHR, FOXE1, LEP, and CRYGD) were deregulated • Transcription of organ-specific genes was suppressed in virus-positive specimens of the ovary, pancreas, and thyroid • Upregulation of interferon-stimulated genes (ISGs)	• Downregulation of two β cell genes coding for insulin and islet amyloid polypeptide (IAPP). • When SARS-CoV-2 detected in tissues, significant transcriptional alterations of endocrine genes were more evidenced.
(122)	Pancreatic samples from individuals who became hyperglycemic patients after COVID-19	• Histopathology • Immunohistochemistry • Immunofluorescence • RT-PCR • Electron Microscopy	• Viral RNA detected • Proinsulin or large number of immature insulin granules observed • Cell death markers such as peripheral unmethylated INS DNA was detected	• Pancreatic β-cells dysfunction such as “β-cell–altered proinsulin processing” and “β-cell degeneration and hyperstimulation” observed.
(40)	Brain, pancreas, lungs, heart, liver and other tissues	• RNA *in situ* (RNAscope)	• Viral RNA and subgenomic RNA in various tissues months after the infection (up to 230 days)	• Diabetic nephropathy observed in some samples
	Human blood			
(187)	Blood of individuals with COVID-19	• A systematic review and meta-analysis	• SARS-CoV-2 RNA and viral fragments	• Higher incidence of new-onset diabetes and hyperglycemia in COVID patients. • Increased risk of new-onset diabetes by about 40% following SARS-CoV-2 infection. • Pancreatic islet cell stress, reduced insulin expression, and β-cell apoptosis, has been observed.
(188)	Blood of individuals with and without T2DM and COVID-19	• Gene Expression Omnibus (GEO) database (GSE95849 and GSE164805) were analyzed to identify differentially expressed genes (DEGs)	• Four upregulated common genes including DHX15, USP14, COPS3, TYK2, and downregulated RIOK2 gene has been identified.	• TYK2 regulates apoptosis in pancreatic islet β-cells and thus may contribute to the progression of diabetes by affecting pancreatic β-cells. • Common feature genes such as DHX15, USP14, COPS3, TYK2, and RIOK2 and their co-regulatory pathways are associated with both T2DM and COVID-19. • Shows bidirectional relationship between SARS-CoV-2 infection and diabetes.
	Animal model			
(189)	Non -human primate model (African green monkey)	• RNAscope, quantitative real-time PCR, blood biochemistry, virologic, and immunologic parameters	• At week 5, gRNA and sgRNA were detected in some animals but not at 18 weeks post infection • Dysregulated blood chemokine signatures such as elevated CCL25 • Immunoglobulin A (IgA) and IgG responses against SARS-CoV-2 spike, S1RBD proteins, and nucleocapsid observed up to 4 months after infection.	• Exhibit persistent early-onset hyperglycemia within four months of post infection. • Several immunologic and metabolic disturbances, such as elevated chemokines (CCL8, CCL19, CCL25 etc.) that are associated with hyperglycemia observed within 18 weeks of post infection. • Elevated level of CCL25 is potentially linked to pancreas GDNF impaired insulin secretion.
(190)	Mice: Wild type mice, C57BL/6J mice, Diabetic C57BL/6J mice with leptin receptor gene deficiency (db/db mice)	• qRT-PCR, RNA sequencing, Histology	• Higher gRNA and sgRNA detected in db/db mice than wild type mice within 7 days of post infection	• A sever islet cell loss and insulin resistance was observed in SARS-CoV-2 infected diabetic mice than uninfected diabetic mice. • Severe diabetes symptoms were noticeable in diabetic mice infected with SARS-CoV-2 than wild type. • The pancreas of SARS-CoV-2 infected db/db mice exhibited a significantly reduced islet cell as compared with that in uninfected db/db mice. • SARS-CoV-2 infection and Diabetes were risk factors for one another.

A systematic review on SARS-CoV-2 RNA persistence in COVID survivors with post-COVID symptoms documented a 5% -59% prevalence of SARS-CoV-2 RNA within the first two months post-infection (194).

In a retrospective autopsy cohort study, the SARS-CoV-2 genome was detected in the pancreas, testis, adrenal gland, thyroid, anterior pituitary, and white adipose tissue (195). Similarly, with different degrees across tissues, viral RNA was detected in liver, lung, breast, skin, kidney, blood vessel, stomach, intestine, brain, and thyroid biopsy samples (44). The persistence of fecal viral RNA up to 230 days post-infection has also been documented (196). S gene RNA was detected in acral sites in patient cohorts from biopsied pernio samples (197). Also, different degrees of spike protein were also identified in the serum of individuals with post-COVID syndrome, patients with myalgic encephalomyelitis, and post-COVID recovered controls within 4–31 months of post SARS-CoV-2 infection (198). Furthermore, both subgenomic RNA and viral RNA were detected in the same solid tissue samples (44). However, viral debris and SARS-CoV-2 RNA persistence in non-pulmonary tissues may vary depending on tissue-specific factors (such as the degree of immune response) and RNA clearance efficiency (40, 199). Viral detection is also influenced by methodology, sample size, and other factors that may contribute to biases in reported results.

Numerous studies have revealed that higher viral copy numbers are strongly associated with the development of long COVID symptoms (44, 193). A cross-sectional cohort study showed significant association between long COVID development and viral RNA detected in recovered patients (44**) (****Table 3**).

Similarly, another cohort study reported significantly higher risk and more symptoms of long COVID in patients with slowed SARS-CoV-2 viral clearance rates during the active phase of infection (200).

Actively replicating viruses were detected in human hepatocytes from postmortem liver biopsies. Moreover, new infectious viral particles were also found in *in vitro* SARS-CoV-2–infected hepatocytes (186). Remarkably, up to two years after initial SARS-CoV-2 infection, studies reported the presence of double-stranded viral RNA, evidencing active viral life cycling (183, 201). Specifically, Menezes et al. employed blood digital transcriptomics and identified nucleocapsid, M^pro^, ORF3a, ORF7a, and antisense ORF1ab RNA in blood samples of long COVID patients (201). A recent study further confirmed that SARS-CoV-2 remains infectious postmortem for 5 days at room temperature (37 °C) and over 7 days at 4 °C (202).

Data are sparse on the prevalence of SARS-CoV-2 persistence at the community level. Among the few studies demonstrating viral load kinetics and evolution of viral dynamics, Ghafari et al. monitored 381 individuals with SARS-CoV-2 RNA using viral sequence data (193). While high viral titers were detected for at least 30 days in the 381 individuals, 54 of them showed viral RNA persisting for at least 60 days (193).

Another central factor in understanding long COVID symptoms is the nature of RNA persistence in different cell types, body sites, and viral variants. For instance, *in vitro*–infected human hepatocytes showed varying susceptibility to different SARS-CoV-2 variants (186).

As some studies reported that Omicron variants appear to cause lower risk of long COVID compared with previous variants (203). However, these variations may be partially attributed to viral load (44), and pre-existing immunity (199).

Given that mutations in accessory genes and other proteins can interfere with innate immune signaling and host machinery, thereby contributing to replication and pathogenesis, the effect of viral mutations on protein persistence is also of interest. Mutations that enhance viral fitness (the ability to spread among individuals (204), mutations also emerge from persistent infections. For example, mutations found in Alpha, BA.1, BA.2, and Delta lineages suggest overall higher fitness (193).

In the molecular “arms race” between host and virus, host microRNAs (miRNAs) attack viral RNAs, while viral miRNAs target host transcripts to facilitate replication. Consequently, the host response determines the type and level of miRNAs expressed in COVID-19 patients (205). Among several reports, a systematic review reported a significant association between COVID-19 and new-onset diabetes (206), suggesting that survivors may be at higher risk of developing new-onset diabetes. Therefore, to establish a potential molecular link between long COVID and new-onset diabetes, it is important to examine gene, protein, and mRNA expression in SARS-CoV-2 patients across diverse sample types and methodologies. For example, endothelial cells release circulating extracellular vesicles containing selected miRNAs (207), such as a specific microRNA signatures—such as miR-34a—have been identified as potential predictors for COVID-19–associated new-onset diabetes (208). Likewise, miRNA signatures linked to macrophage activation have been widely implicated in predicting COVID-19 severity in T2DM patients (209).

## 5 Conclusions and future perspectives

As herein discussed, there are pathobiological mechanistic links between SARS-CoV-2 infection and new-onset DM. A considerable number of current studies document that COVID-19 survivors face increased risks of new-onset DM and related metabolic disorders. With increasing risk of diabetes over time post-infection, studies have shown higher prevalence of T2DM compared with T1DM. Given the complex nature of diabetes and the multisystem effects of long COVID, studies that reveal the molecular mechanisms remain limited thus far. Despite the fact that in some cases it may be challenging to “rule out” that the incidence of diabetes is solely due to SARS-CoV-2 infection, the potential mechanisms include direct damage to pancreatic β-cells, inflammation, insulin resistance, autoimmune responses, among others.

Dysregulated ACE2/RAS pathways have been tied to multiple inter-organ pathogenesis. Receptor-mediated viral entry could lead to altered ACE2/RAS signaling, followed by subsequent local and systemic tissue damage, including inflammatory responses, oxidative stress, mitochondrial dysfunction, gut microbiome dysbiosis, endothelial dysfunction, and vascular complications.

Such complex inter-organ and inter-tissue pathogenesis—including the perturbed RAS pathway in PASC and long-term outcomes of long COVID—is accompanied by inflammatory pathways such as leukocyte infiltration and increased inflammatory cytokines. These immune perturbations could mediate dysregulation of IRFs (such as overexpression of IRF1) as a main cellular/molecular mechanistic link to a multitude of inter-organ damages and metabolic alterations (178).

Impaired glucose homeostasis or hyperglycemia—whether due to low insulin production or secretion (from pancreatic damage) or insulin resistance (impaired glucose metabolism by other organs/tissues such as liver, adipose tissue, and skeletal muscle)—could contribute to the progression of both new-onset T1DM and T2DM. It is also important to note that acute inflammatory responses, medications such as glucocorticoids, and cytokine storm–related stress may elicit transient hyperglycemia. This should be clearly defined to better understand the long-term contribution of hyperglycemia to the progression of diabetes. Furthermore, SARS-CoV-2 can induce reprogramming of host glucose metabolism. For instance, Rochowski et al. reported that without any noticeable effects on the pancreas, SARS-CoV-2 infection increased blood glucose concentration and cardiopulmonary GLUT expression through an AMPK-dependent mechanism (210).

The presence of viral RNA or fragments could directly damage different islets of the pancreas, contributing to insulin resistance and β-cell dysfunction, which in turn may lead to progression of new-onset diabetes. Although it is well documented that SARS-CoV-2 persists in several tissues for months to years, mechanisms of viral persistence are not yet fully understood, highlighting the need for large human studies on SARS-CoV-2 reservoirs and related biological factors in PASC. Such studies will enable identification of disease mechanisms—including progression of new-onset DM—biomarkers, and potential therapeutics for other chronic conditions increasingly linked to persistent SARS-CoV-2 infection (211, 212).

Several findings and systematic review studies suggest that systemic inflammation and immune-mediated changes reflected in biomarkers are important contributors to the development of new-onset diabetes in COVID-19 patients (213–215). For instance, recent cohort study in individuals with long COVID in Sweden and the United Kingdom identified soluble markers of systemic inflammation and immune dysregulation that correlate with metabolic complications such as diabetes (216).

Despite numerous challenges, there is a pressing need for biomarker and therapeutic targets for PASC clinical trials. One central question in long COVID biomarker development is “biomarkers for what?” A major challenge in biomarker discovery is defining the specific symptoms or biological changes to be measured, given that long COVID is a complex multisystem disorder affecting nearly all organs. In addition, difficulties in identifying accepted biomarkers present another major obstacle. It is also worth noting that biomarker patterns may differ between individuals who fully recover and those who show persistent PASC symptoms. The lack of a biomarker that strongly associates with long COVID may also limit the interpretation of many study results. Despite these challenges, several systematic reviews and meta-analyses have reported potential biomarkers warranting further investigation. For example, Lai et al. systematically reviewed 28 studies and 113 blood biomarkers significantly associated with long COVID, categorizing them into acute-phase proteins, biochemical markers, cytokines/chemokines, neurological markers, vascular markers, and other biomarkers (217). More broadly, in PASC clinical trials, biomarkers may be stratified as biomarkers for improved diagnosis, primary outcome measures, or therapeutic candidates. Likewise, direct-acting and host-directed antivirals and immunomodulators (such as interferons and monoclonal antibodies) have been proposed as potential therapeutics for treatment of SARS-CoV-2 reservoirs in PASC (211, 218). Furthermore, Peluso and Deeks hypothesized several categories of biomarkers for long COVID—mechanistic, diagnostic, predictive, and surrogate biomarker (22); they articulated that the identification of surrogate markers would rapidly accelerate therapeutic development.

Overall, a better understanding of the crosstalk between host cell metabolism and SARS-CoV-2 in long COVID patients could accelerate the discovery of biomarkers as well as therapeutic options targeting new-onset diabetes and related metabolic complications.
