# Supplementary material for: Molecular analysis of long COVID and new-onset diabetes mellitus: pathobiological relationships and current mechanistic views
Source: Front Endocrinol (Lausanne). 2025 Dec 18;16:1737894. doi: 10.3389/fendo.2025.1737894 (PMC12756089; doi:10.3389/fendo.2025.1737894)
Supplement: Supplementary file 1 [file Table1.docx]

**Supplementary Table 1.** Summary of SARS-CoV-2 replicase proteins and their functions.

| Non-structural protein | Function | Ref. |
| --- | --- | --- |
| Nsp1 | - Host shut-off factor: binds host ribosome and leads to host mRNA degradation and host translation shut-off - Stimulates viral RNA translation | (Wang et al., 2023, Schubert et al., 2020) |
| Nsp2 | - Act as a potential delivery vehicle for proteins - Stimulates viral RNA translation | (Zheng et al., 2024, Korneeva et al., 2023) |
| Nsp3 | - Play a multifaceted role: binds RNA, and nucleocapsid protein via the amino-terminal Ubl domain, mono-ADP-ribosylhydrolase, PLpro, generates RNA-synthesizing double-membrane vesicles or replication organelle formation - Key component of molecular pore spanning in double-membrane vesicles | (Alhammad et al., 2021, Wolff et al., 2020, Zimmermann et al., 2023, Cong et al., 2020, Bessa et al., 2022, Shin et al., 2020) |
| Nsp4 | - Replication organelle formation (double-membrane vesicles) | (Ricciardi et al., 2022, Zimmermann et al., 2023) |
| Nsp5 | - Picornavirus 3C-like protease or main protease (Mpro) - Promotes downregulation of major histocompatibility complex (MHC) II by targeting histone deacetylase 2 (HDAC2) - Downregulates antiviral stress granule formation | (Taefehshokr et al., 2024, Zheng et al., 2022, Yadav et al., 2022, Meyer et al., 2021) |
| Nsp6 | - Organize the double-membrane vesicle complexes and assists replication organelle formation | (Ricciardi et al., 2022) |
| Nsp7 | - Subunit (cofactor) of the RNA-dependent RNA polymerase (RdRp) holoenzyme | (Yan et al., 2021a) |
| Nsp8 | - Putative primase, and also acts as putative 3′-terminal adenylyltransferase - Subunit and mediates RdRp complex (Nsp12-Nsp8-Nsp7): stimulates Nsp14-Nsp10 activity | (Baddock et al., 2022, Kirchdoerfer and Ward, 2019) |
| Nsp9 | - Serves as a molecular connector in the RTC: Putative RNA-binding protein, NiRAN UMP-transferase substrate - Disrupts host antiviral pathways | (Lundrigan et al., 2024, Yan et al., 2021b) |
| Nsp10 | - Acts as a scaffolding and stimulatory cofactor: maintains the Nsp14 exonuclease and Nsp16 methyltransferase activities - Play a role in modulating ribosomal frameshifting process | (Ma et al., 2015, Bouvet et al., 2014, Bhatt et al., 2021) |
| Nsp11 | - Unknown |  |
| Nsp12 | - Involved in RNA capping via NiRAN domain: NMPylase, catalyze the addition of nucleoside monophosphate (NMP) to nsp9. | (Slanina et al., 2021, Yan et al., 2021a, Walker et al., 2021) |
| Nsp13 | - Serves as a bifunctional enzyme; RNA helicase, and RNA 5′-phosphatase - Disrupts RNA-protein interaction - Functions as interferon antagonist | (Yuen et al., 2020, Sommers et al., 2023, Lawal et al., 2024, Yan et al., 2021a) |
| Nsp14 | - Involved in host protein synthesis shutdown by acting as translation inhibitory factor - Proofreading 3′-5′ exoribonuclease, N7-methyltransferase | (Hsu et al., 2021, Moeller et al., 2022, Yan et al., 2021b, Lin et al., 2021) |
| Nsp15 | - Interferon antagonist via poly-Uridylate-specific endonuclease domain (EndoU) | (Otter et al., 2024, Zhang et al., 2023) |
| Nsp16 | - Viral mRNA capping via 2′-O-Methyltransferase - Disrupts host’s splicing machinery by targeting snRNA | (Viswanathan et al., 2020, Dias et al., 2024, Banerjee et al., 2020) |

**References**

ALHAMMAD, Y. M., KASHIPATHY, M. M., ROY, A., GAGNÉ, J.-P., MCDONALD, P., GAO, P., NONFOUX, L., BATTAILE, K. P., JOHNSON, D. K. & HOLMSTROM, E. D. 2021. The SARS-CoV-2 conserved macrodomain is a mono-ADP-ribosylhydrolase. *Journal of virology,* 95**,** 10.1128/jvi. 01969-20.

BADDOCK, H. T., BROLIH, S., YOSAATMADJA, Y., RATNAWEERA, M., BIELINSKI, M., SWIFT, L. P., CRUZ-MIGONI, A., FAN, H., KEOWN, J. R. & WALKER, A. P. 2022. Characterization of the SARS-CoV-2 ExoN (nsp14ExoN–nsp10) complex: implications for its role in viral genome stability and inhibitor identification. *Nucleic acids research,* 50**,** 1484-1500.

BANERJEE, A. K., BLANCO, M. R., BRUCE, E. A., HONSON, D. D., CHEN, L. M., CHOW, A., BHAT, P., OLLIKAINEN, N., QUINODOZ, S. A. & LONEY, C. 2020. SARS-CoV-2 disrupts splicing, translation, and protein trafficking to suppress host defenses. *Cell,* 183**,** 1325-1339. e21.

BESSA, L. M., GUSEVA, S., CAMACHO-ZARCO, A. R., SALVI, N., MAURIN, D., PEREZ, L. M., BOTOVA, M., MALKI, A., NANAO, M. & JENSEN, M. R. 2022. The intrinsically disordered SARS-CoV-2 nucleoprotein in dynamic complex with its viral partner nsp3a. *Science advances,* 8**,** eabm4034.

BHATT, P. R., SCAIOLA, A., LOUGHRAN, G., LEIBUNDGUT, M., KRATZEL, A., MEURS, R., DREOS, R., O’CONNOR, K. M., MCMILLAN, A. & BODE, J. W. 2021. Structural basis of ribosomal frameshifting during translation of the SARS-CoV-2 RNA genome. *Science,* 372**,** 1306-1313.

BOUVET, M., LUGARI, A., POSTHUMA, C. C., ZEVENHOVEN, J. C., BERNARD, S., BETZI, S., IMBERT, I., CANARD, B., GUILLEMOT, J.-C. & LÉCINE, P. 2014. Coronavirus Nsp10, a critical co-factor for activation of multiple replicative enzymes. *Journal of Biological Chemistry,* 289**,** 25783-25796.

CONG, Y., ULASLI, M., SCHEPERS, H., MAUTHE, M., V’KOVSKI, P., KRIEGENBURG, F., THIEL, V., DE HAAN, C. A. & REGGIORI, F. 2020. Nucleocapsid protein recruitment to replication-transcription complexes plays a crucial role in coronaviral life cycle. *Journal of virology,* 94**,** 10.1128/jvi. 01925-19.

DIAS, T. L., MAMEDE, I., DE TOLEDO, N. E., QUEIROZ, L. R., CASTRO, Í., POLIDORO, R., DEL-BEM, L. E., NAKAYA, H. & FRANCO, G. R. 2024. SARS-CoV-2 Selectively Induces the Expression of Unproductive Splicing Isoforms of Interferon, Class I MHC, and Splicing Machinery Genes. *International Journal of Molecular Sciences,* 25**,** 5671.

HSU, J. C.-C., LAURENT-ROLLE, M., PAWLAK, J. B., WILEN, C. B. & CRESSWELL, P. 2021. Translational shutdown and evasion of the innate immune response by SARS-CoV-2 NSP14 protein. *Proceedings of the National Academy of Sciences,* 118**,** e2101161118.

KIRCHDOERFER, R. N. & WARD, A. B. 2019. Structure of the SARS-CoV nsp12 polymerase bound to nsp7 and nsp8 co-factors. *Nature communications,* 10**,** 2342.

KORNEEVA, N., KHALIL, M. I., GHOSH, I., FAN, R., ARNOLD, T. & DE BENEDETTI, A. 2023. SARS-CoV-2 viral protein Nsp2 stimulates translation under normal and hypoxic conditions. *Virology Journal,* 20**,** 55.

LAWAL, M. M., ROY, P. & MCCULLAGH, M. 2024. Role of ATP hydrolysis and product release in the translocation mechanism of SARS-CoV-2 NSP13. *The Journal of Physical Chemistry B,* 128**,** 492-503.

LIN, S., CHEN, H., CHEN, Z., YANG, F., YE, F., ZHENG, Y., YANG, J., LIN, X., SUN, H. & WANG, L. 2021. Crystal structure of SARS-CoV-2 nsp10 bound to nsp14-ExoN domain reveals an exoribonuclease with both structural and functional integrity. *Nucleic acids research,* 49**,** 5382-5392.

LUNDRIGAN, E., TOUDIC, C., PENNOCK, E. & PEZACKI, J. P. 2024. SARS-CoV-2 Protein Nsp9 Is Involved in Viral Evasion through Interactions with Innate Immune Pathways. *ACS Omega*.

MA, Y., WU, L., SHAW, N., GAO, Y., WANG, J., SUN, Y., LOU, Z., YAN, L., ZHANG, R. & RAO, Z. 2015. Structural basis and functional analysis of the SARS coronavirus nsp14–nsp10 complex. *Proceedings of the National Academy of Sciences,* 112**,** 9436-9441.

MEYER, B., CHIARAVALLI, J., GELLENONCOURT, S., BROWNRIDGE, P., BRYNE, D. P., DALY, L. A., GRAUSLYS, A., WALTER, M., AGOU, F. & CHAKRABARTI, L. A. 2021. Characterising proteolysis during SARS-CoV-2 infection identifies viral cleavage sites and cellular targets with therapeutic potential. *Nature Communications,* 12**,** 5553.

MOELLER, N. H., SHI, K., DEMIR, Ö., BELICA, C., BANERJEE, S., YIN, L., DURFEE, C., AMARO, R. E. & AIHARA, H. 2022. Structure and dynamics of SARS-CoV-2 proofreading exoribonuclease ExoN. *Proceedings of the National Academy of Sciences,* 119**,** e2106379119.

OTTER, C. J., BRACCI, N., PARENTI, N. A., YE, C., ASTHANA, A., BLOMQVIST, E. K., TAN, L. H., PFANNENSTIEL, J. J., JACKSON, N. & FEHR, A. R. 2024. SARS-CoV-2 nsp15 endoribonuclease antagonizes dsRNA-induced antiviral signaling. *Proceedings of the National Academy of Sciences,* 121**,** e2320194121.

RICCIARDI, S., GUARINO, A. M., GIAQUINTO, L., POLISHCHUK, E. V., SANTORO, M., DI TULLIO, G., WILSON, C., PANARIELLO, F., SOARES, V. C. & DIAS, S. S. 2022. The role of NSP6 in the biogenesis of the SARS-CoV-2 replication organelle. *Nature,* 606**,** 761-768.

SCHUBERT, K., KAROUSIS, E. D., JOMAA, A., SCAIOLA, A., ECHEVERRIA, B., GURZELER, L.-A., LEIBUNDGUT, M., THIEL, V., MÜHLEMANN, O. & BAN, N. 2020. SARS-CoV-2 Nsp1 binds the ribosomal mRNA channel to inhibit translation. *Nature Structural & Molecular Biology,* 27**,** 959-966.

SHIN, D., MUKHERJEE, R., GREWE, D., BOJKOVA, D., BAEK, K., BHATTACHARYA, A., SCHULZ, L., WIDERA, M., MEHDIPOUR, A. R. & TASCHER, G. 2020. Papain-like protease regulates SARS-CoV-2 viral spread and innate immunity. *Nature,* 587**,** 657-662.

SLANINA, H., MADHUGIRI, R., BYLAPUDI, G., SCHULTHEIß, K., KARL, N., GULYAEVA, A., GORBALENYA, A. E., LINNE, U. & ZIEBUHR, J. 2021. Coronavirus replication–transcription complex: Vital and selective NMPylation of a conserved site in nsp9 by the NiRAN-RdRp subunit. *Proceedings of the National Academy of Sciences,* 118**,** e2022310118.

SOMMERS, J. A., LOFTUS, L. N., JONES, M. P., LEE, R. A., HAREN, C. E., DUMM, A. J. & BROSH, R. M. 2023. Biochemical analysis of SARS-CoV-2 Nsp13 helicase implicated in COVID-19 and factors that regulate its catalytic functions. *Journal of Biological Chemistry,* 299.

TAEFEHSHOKR, N., LAC, A., VRIEZE, A. M., DICKSON, B. H., GUO, P. N., JUNG, C., BLYTHE, E. N., FINK, C., AKTAR, A. & DIKEAKOS, J. D. 2024. SARS-CoV-2 NSP5 antagonizes MHC II expression by subverting histone deacetylase 2. *Journal of Cell Science,* 137.

VISWANATHAN, T., ARYA, S., CHAN, S.-H., QI, S., DAI, N., MISRA, A., PARK, J.-G., OLADUNNI, F., KOVALSKYY, D. & HROMAS, R. A. 2020. Structural basis of RNA cap modification by SARS-CoV-2. *Nature communications,* 11**,** 3718.

WALKER, A. P., FAN, H., KEOWN, J. R., KNIGHT, M. L., GRIMES, J. M. & FODOR, E. 2021. The SARS-CoV-2 RNA polymerase is a viral RNA capping enzyme. *Nucleic acids research,* 49**,** 13019-13030.

WANG, Y., KIRKPATRICK, J., LAGE, S. Z. & CARLOMAGNO, T. 2023. Structural insights into the activity regulation of full-length non-structural protein 1 from SARS-CoV-2. *Structure,* 31**,** 128-137.e5.

WOLFF, G., LIMPENS, R. W., ZEVENHOVEN-DOBBE, J. C., LAUGKS, U., ZHENG, S., DE JONG, A. W., KONING, R. I., AGARD, D. A., GRÜNEWALD, K. & KOSTER, A. J. 2020. A molecular pore spans the double membrane of the coronavirus replication organelle. *Science,* 369**,** 1395-1398.

YADAV, R., COUROUBLE, V. V., DEY, S. K., HARRISON, J. J. E., TIMM, J., HOPKINS, J. B., SLACK, R. L., SARAFIANOS, S. G., RUIZ, F. X. & GRIFFIN, P. R. 2022. Biochemical and structural insights into SARS-CoV-2 polyprotein processing by Mpro. *Science Advances,* 8**,** eadd2191.

YAN, L., GE, J., ZHENG, L., ZHANG, Y., GAO, Y., WANG, T., HUANG, Y., YANG, Y., GAO, S. & LI, M. 2021a. Cryo-EM structure of an extended SARS-CoV-2 replication and transcription complex reveals an intermediate state in cap synthesis. *Cell,* 184**,** 184-193. e10.

YAN, L., YANG, Y., LI, M., ZHANG, Y., ZHENG, L., GE, J., HUANG, Y. C., LIU, Z., WANG, T. & GAO, S. 2021b. Coupling of N7-methyltransferase and 3′-5′ exoribonuclease with SARS-CoV-2 polymerase reveals mechanisms for capping and proofreading. *Cell,* 184**,** 3474-3485. e11.

YUEN, C.-K., LAM, J.-Y., WONG, W.-M., MAK, L.-F., WANG, X., CHU, H., CAI, J.-P., JIN, D.-Y., TO, K. K.-W. & CHAN, J. F.-W. 2020. SARS-CoV-2 nsp13, nsp14, nsp15 and orf6 function as potent interferon antagonists. *Emerging microbes & infections,* 9**,** 1418-1428.

ZHANG, D., JI, L., CHEN, X., HE, Y., SUN, Y., JI, L., ZHANG, T., SHEN, Q., WANG, X. & WANG, Y. 2023. SARS-CoV-2 Nsp15 suppresses type I interferon production by inhibiting IRF3 phosphorylation and nuclear translocation. *Iscience,* 26.

ZHENG, N., LIU, S., CHEN, J., XU, Y., CAO, W., LIN, J., LU, G. & ZHANG, G. 2024. SARS-CoV-2 NSP2 as a Potential Delivery Vehicle for Proteins. *Molecular Pharmaceutics,* 21**,** 1149-1159.

ZHENG, Y., DENG, J., HAN, L., ZHUANG, M.-W., XU, Y., ZHANG, J., NAN, M.-L., XIAO, Y., ZHAN, P. & LIU, X. 2022. SARS-CoV-2 NSP5 and N protein counteract the RIG-I signaling pathway by suppressing the formation of stress granules. *Signal transduction and targeted therapy,* 7**,** 22.

ZIMMERMANN, L., ZHAO, X., MAKROCZYOVA, J., WACHSMUTH-MELM, M., PRASAD, V., HENSEL, Z., BARTENSCHLAGER, R. & CHLANDA, P. 2023. SARS-CoV-2 nsp3 and nsp4 are minimal constituents of a pore spanning replication organelle. *Nature Communications,* 14**,** 7894.
